# Supplementary material for: Genome-wide association analysis provides insights into the molecular etiology of dilated cardiomyopathy
Source: Nat Genet. 2024 Nov 21;56(12):2646–58. doi: 10.1038/s41588-024-01952-y (PMC11631752; doi:10.1038/s41588-024-01952-y)
Supplement: Supplementary file 1 — Supplementary Information 1–7 and References. [file 41588_2024_1952_MOESM1_ESM.pdf]

nature genetics

Supplementary information https://doi.org/10.1038/s41588-024-01952-y

Genome-wide association analysis provides insights into the molecular etiology of dilated cardiomyopathy

In the format provided by the authors and unedited

Supplementary Information

1. STUDY DEFINITIONS FOR PHENOTYPING OF CASES AND CONTROLS 2

2. INDIVIDUAL STUDY COHORT DESCRIPTIONS AND ETHICS APPROVALS 10

3. STUDY LEVEL GWAS METHODS AND QUALITY CONTROL 15

4. MENDELIAN CARDIOMYOPATHY CAUSING GENES FROM CLINGEN AND GENCC 16

5. CURATION OF PATHOGENIC AND LIKELY PATHOGENIC RARE VARIANTS IN DCM-CAUSING GENES IN THE UK BIOBANK 17

6. INDIVIDUAL STUDY ACKNOWLEDGEMENTS 18

7. CONSORTIA MEMBERS 21

SUPPLEMENTARY INFORMATION REFERENCES 33

1. Study definitions for phenotyping of cases and controls

The primary GWAS phenotype of DCM required the presence of LV systolic dysfunction (LVSD) in the absence of coronary artery disease, valvular heart disease, or congenital heart disease (see below). Participants without heart failure, coronary artery disease, valvular heart disease, or congenital heart disease were selected as controls.

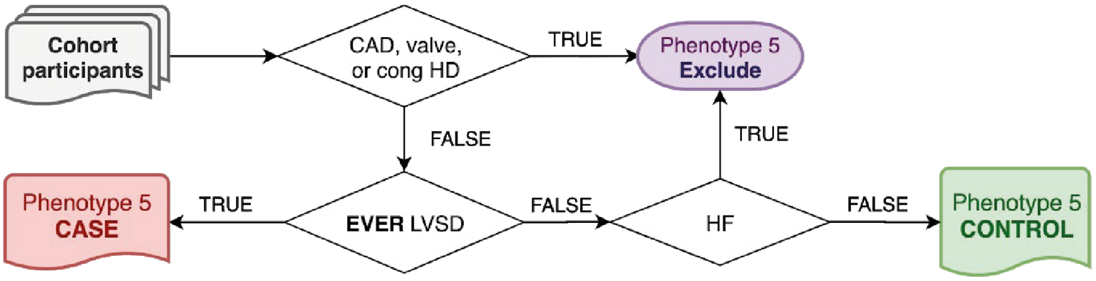

A stricter DCM diagnosis was used for sensitivity analysis and required a clinical diagnosis of DCM based on clinical guidelines, or documented ICD-10 code (I42.0) in the absence of coronary artery, valvular heart disease, or congenital heart disease codes (see below).

LVSD:

- LVEF <50% on cardiac imaging
- Manually adjudicated diagnosis of heart failure with reduced ejection fraction (HFrEF), heart failure with mid-range ejection fraction HFmrEF, systolic heart failure, DCM, permanent pacemaker implantation, arrhythmogenic cardiomyopathy, inflammatory cardiomyopathy and chemotherapy-related cardiomyopathy.
- >1 occurrence of the following codes:

Source	Code	Description
ICD10	I42.0	Dilated cardiomyopathy (Congestive cardiomyopathy)
ICD10	I42.6	Alcoholic cardiomyopathy
ICD10	I42.7	Cardiomyopathy due to drug and external agent
ICD10	I25.5	Ischaemic cardiomyopathy
ICD10	I50.2	Systolic (congestive) heart failure
ICD10	O90.3	Cardiomyopathy in the puerperium
ICD9	428.2	Systolic heart failure
ICD9	674.5	Peripartum cardiomyopathy
ICD9	425.5	Alcoholic cardiomyopathy
ICD9	425.9	Secondary cardiomyopathy, unspecified
OPCS4	K61.7	Cardiac resynchronisation device
CPT4	33224	Insertion of pacing electrode, cardiac venous system, for left ventricular pacing, with attachment to previously placed pacemaker or implantable defibrillator pulse generator (including revision of pocket, removal, insertion, and/or replacement of existing generator)
CPT4	33225	Insertion of pacing electrode, cardiac venous system, for left ventricular pacing, at time of insertion of implantable defibrillator or pacemaker pulse generator (e.g., for upgrade to dual chamber system) (List separately in addition to code for primary procedure)

CPT4	33226	Repositioning of previously implanted cardiac venous system (left ventricular) electrode (including removal, insertion and/or replacement of existing generator)

Coronary artery disease (CAD), valvular heart disease (VHD) or congenital heart disease (CHD):

- Manually adjudicated diagnosis of myocardial infarction or revascularization.
- Severe primary valve disease defined by a relevant surgical or percutaneous valve procedure.
- Any congenital malformation of the heart or great vessels defined by diagnosis or relevant surgical or percutaneous procedure.
- Any rheumatic heart valve disease defined by diagnosis.
- >1 occurrence of the following codes:

Source	Code	Description	Phenotype class
ICD10	I21.*	Acute myocardial infarction	CAD
ICD10	I22.*	Subsequent myocardial infarction	CAD
ICD10	I23.*	Certain current complications following acute myocardial infarction	CAD
ICD10	I24.1	Dressler's syndrome	CAD
ICD10	I25.2	Old myocardial infarction	CAD
ICD10	I25.5	Ischaemic cardiomyopathy	CAD
ICD10	I25.6	Silent myocardial ischaemia	CAD
ICD10	745.*	Bulbus cordis anomalies and anomalies of cardiac septal closure	CHD
ICD10	Q20.*	Congenital malformations of cardiac chambers and connections	CHD
ICD10	Q21.*	Congenital malformations of cardiac septa	CHD
ICD10	Q22.*	Congenital malformations of pulmonary and tricuspid valves	CHD
ICD10	Q23.*	Congenital malformations of aortic and mitral valves	CHD
ICD10	Q24.*	Other congenital malformations of heart	CHD
ICD10	Q25.*	Congenital malformations of great arteries	CHD
ICD10	Q26.*	Congenital malformations of great veins	CHD
ICD10	746.*	Other congenital anomalies of heart	VHD
ICD10	I05.*	Rheumatic mitral valve diseases	VHD
ICD10	I06.*	Rheumatic aortic valve diseases	VHD
ICD10	I07.*	Rheumatic tricuspid valve diseases	VHD
ICD9	410.*	Myocardial infarction	CAD
ICD9	412.*	Old myocardial infarction	CAD
ICD9	394.*	Diseases of mitral valve	VHD
ICD9	395.*	Diseases of aortic valve	VHD
ICD9	396.*	Diseases of mitral and aortic valves	VHD
ICD9	397.*	Diseases of other endocardial structures	VHD
OPCS4	K40.*	Saphenous vein graft replacement of coronary artery	CAD
OPCS4	K41.*	Other autograft replacement of coronary artery	CAD
OPCS4	K42.*	Allograft replacement of coronary artery	CAD
OPCS4	K43.*	Prosthetic replacement of coronary artery	CAD
OPCS4	K44.*	Other replacement of coronary artery	CAD
OPCS4	K45.*	Connection of thoracic artery to coronary artery	CAD
OPCS4	K46.*	Other bypass of coronary artery	CAD

OPCS4	K47.*	Repair of coronary artery	CAD
OPCS4	K48.*	Other open operations on coronary artery	CAD
OPCS4	K49.*	Transluminal balloon angioplasty of coronary artery	CAD
OPCS4	K50.*	Other therapeutic transluminal operations on coronary artery	CAD
OPCS4	K75.*	Percutaneous transluminal balloon angioplasty and insertion of stent into coronary artery	CAD
OPCS4	K25.*	Plastic repair of mitral valve	VHD
OPCS4	K26.*	Plastic repair of aortic valve	VHD
OPCS4	K27.*	Plastic repair of tricuspid valve	VHD
OPCS4	K28.*	Plastic repair of pulmonary valve	VHD
OPCS4	K29.*	Plastic repair of unspecified valve of heart	VHD
OPCS4	K30.*	Revision of plastic repair of valve of heart	VHD
OPCS4	K31.*	Open incision of valve of heart	VHD
OPCS4	K32.*	Closed incision of valve of heart	VHD
OPCS4	K34.*	Other open operations on valve of heart	VHD
OPCS4	K36.*	Excision of valve of heart	VHD
OPCS4	K37.*	Removal of obstruction from structure adjacent to valve of heart	VHD
OPCS4	K38.*	Other operations on structure adjacent to valve of heart	VHD
CPT4	33510	Coronary artery bypass, vein only; single coronary venous graft	CAD
CPT4	33510	Coronary artery bypass, vein only; single coronary venous graft	CAD
CPT4	33511	Coronary artery bypass, vein only; 2 coronary venous grafts	CAD
CPT4	33511	Coronary artery bypass, vein only; 2 coronary venous grafts	CAD
CPT4	33512	Coronary artery bypass, vein only; 3 coronary venous grafts	CAD
CPT4	33512	Coronary artery bypass, vein only; 3 coronary venous grafts	CAD
CPT4	33513	Coronary artery bypass, vein only; 4 coronary venous grafts	CAD
CPT4	33513	Coronary artery bypass, vein only; 4 coronary venous grafts	CAD
CPT4	33530	Reoperation, coronary artery bypass procedure or valve procedure, more than 1 month after original operation (List separately in addition to code for primary procedure)	CAD
CPT4	33533	Coronary artery bypass, using arterial graft(s); single arterial graft	CAD
CPT4	33533	Coronary artery bypass, using arterial graft(s); single arterial graft	CAD
CPT4	33534	Coronary artery bypass, using arterial graft(s); 2 coronary arterial grafts	CAD
CPT4	33534	Coronary artery bypass, using arterial graft(s); 2 coronary arterial grafts	CAD
CPT4	33535	Coronary artery bypass, using arterial graft(s); 3 coronary arterial grafts	CAD
CPT4	33535	Coronary artery bypass, using arterial graft(s); 3 coronary arterial grafts	CAD
CPT4	33536	Coronary artery bypass, using arterial graft(s); 4 or more coronary arterial grafts	CAD
CPT4	33536	Coronary artery bypass, using arterial graft(s); 4 or more coronary arterial grafts	CAD
CPT4	92920	Percutaneous transluminal coronary angioplasty; single major coronary artery or branch	CAD
CPT4	92920	Percutaneous transluminal coronary angioplasty; single major coronary artery or branch	CAD
CPT4	92921	Percutaneous transluminal coronary angioplasty; each additional branch of a major coronary artery	CAD
CPT4	92921	Percutaneous transluminal coronary angioplasty; each additional branch of a major coronary artery	CAD
CPT4	92924	Percutaneous transluminal coronary atherectomy, with coronary angioplasty when performed; single major coronary artery or branch	CAD

CPT4	92924	Percutaneous transluminal coronary atherectomy, with coronary angioplasty when performed; single major coronary artery or branch	CAD
CPT4	92925	Percutaneous transluminal coronary atherectomy, with coronary angioplasty when performed; each additional branch of a major coronary artery	CAD
CPT4	92925	Percutaneous transluminal coronary atherectomy, with coronary angioplasty when performed; each additional branch of a major coronary artery	CAD
CPT4	92928	Percutaneous transcatheter placement of intracoronary stent(s), with coronary angioplasty when performed; single major coronary artery or branch	CAD
CPT4	92928	Percutaneous transcatheter placement of intracoronary stent(s), with coronary angioplasty when performed; single major coronary artery or branch	CAD
CPT4	92929	Percutaneous transcatheter placement of intracoronary stent(s), with coronary angioplasty when performed; each additional branch of a major coronary artery	CAD
CPT4	92929	Percutaneous transcatheter placement of intracoronary stent(s), with coronary angioplasty when performed; each additional branch of a major coronary artery	CAD
CPT4	92933	Percutaneous transluminal coronary atherectomy, with intracoronary stent, with coronary angioplasty when performed; single major coronary artery or branch	CAD
CPT4	92933	Percutaneous transluminal coronary atherectomy, with intracoronary stent, with coronary angioplasty when performed; single major coronary artery or branch	CAD
CPT4	92934	Percutaneous transluminal coronary atherectomy, with intracoronary stent, with coronary angioplasty when performed; each additional branch of a major coronary artery	CAD
CPT4	92934	Percutaneous transluminal coronary atherectomy, with intracoronary stent, with coronary angioplasty when performed; each additional branch of a major coronary artery	CAD
CPT4	92937	Percutaneous transluminal revascularization of or through coronary artery bypass graft (internal mammary, free arterial, venous), any combination of intracoronary stent, atherectomy and angioplasty, including distal protection when performed; single vessel	CAD
CPT4	92937	Percutaneous transluminal revascularization of or through coronary artery bypass graft (internal mammary, free arterial, venous), any combination of intracoronary stent, atherectomy and angioplasty, including distal protection when performed; single vessel	CAD
CPT4	92938	Percutaneous transluminal revascularization of or through coronary artery bypass graft (internal mammary, free arterial, venous), any combination of intracoronary stent, atherectomy and angioplasty, including distal protection when performed; each additional branch subtended by the bypass graft	CAD
CPT4	92938	Percutaneous transluminal revascularization of or through coronary artery bypass graft (internal mammary, free arterial, venous), any combination of intracoronary stent, atherectomy and angioplasty, including distal protection when performed; each additional branch subtended by the bypass graft	CAD
CPT4	92941	Percutaneous transluminal revascularization of acute total/subtotal occlusion during acute myocardial infarction, coronary artery or coronary artery bypass graft, any combination of intracoronary stent, atherectomy and angioplasty, including aspiration thrombectomy when performed, single vessel	CAD
CPT4	92941	Percutaneous transluminal revascularization of acute total/subtotal occlusion during acute myocardial infarction, coronary artery or coronary artery bypass graft, any combination of intracoronary stent, atherectomy and angioplasty, including aspiration thrombectomy when performed, single vessel	CAD
CPT4	92943	Percutaneous transluminal revascularization of chronic total occlusion, coronary artery, coronary artery branch, or coronary artery bypass graft, any combination of intracoronary stent, atherectomy and angioplasty; single vessel	CAD
CPT4	92943	Percutaneous transluminal revascularization of chronic total occlusion, coronary artery, coronary artery branch, or coronary artery bypass graft, any combination of intracoronary stent, atherectomy and angioplasty; single vessel	CAD
CPT4	92944	Percutaneous transluminal revascularization of chronic total occlusion, coronary artery, coronary artery branch, or coronary artery bypass graft, any combination of intracoronary stent, atherectomy and angioplasty; each additional coronary artery, coronary artery branch, or bypass graft	CAD
CPT4	92944	Percutaneous transluminal revascularization of chronic total occlusion, coronary artery, coronary artery branch, or coronary artery bypass graft, any combination of intracoronary	CAD

		stent, atherectomy and angioplasty; each additional coronary artery, coronary artery branch, or bypass graft	
CPT4	92973	Percutaneous transluminal coronary thrombectomy, mechanical*	CAD
CPT4	92973	Percutaneous transluminal coronary thrombectomy, mechanical*	CAD
CPT4	92975	Thrombolysis, coronary; by intracoronary infusion, including selective coronary angiography	CAD
CPT4	92975	Thrombolysis, coronary; by intracoronary infusion, including selective coronary angiography	CAD
CPT4	33608	Repair of complex cardiac anomaly other than pulmonary atresia with ventricular septal defect by construction or replacement of conduit from right or left ventricle to pulmonary artery	CHD
CPT4	33610	Repair of complex cardiac anomalies (eg, single ventricle with subaortic obstruction) by surgical enlargement of ventricular septal defect	CHD
CPT4	33611	Repair of double outlet right ventricle with intraventricular tunnel repair;	CHD
CPT4	33612	Repair of double outlet right ventricle with intraventricular tunnel repair; with repair of right ventricular outflow tract obstruction	CHD
CPT4	33615	Repair of complex cardiac anomalies (eg, tricuspid atresia) by closure of atrial septal defect and anastomosis of atria or vena cava to pulmonary artery (simple Fontan procedure)	CHD
CPT4	33617	Repair of complex cardiac anomalies (e.g., single ventricle by modified Fontan)	CHD
CPT4	33619	Repair of single ventricle with aortic outflow obstruction and aortic arch hypoplasia (hypoplastic left heart syndrome) (eg, Norwood procedure)	CHD
CPT4	33641	Repair atrial septal defect, secundum, with cardiopulmonary bypass, with or without patch	CHD
CPT4	33645	Direct or patch closure, sinus venosus, with or without anomalous pulmonary venous drainage	CHD
CPT4	33647	Repair of atrial septal defect and ventricular septal defect, with direct or patch closure	CHD
CPT4	33660	Repair of incomplete or partial atrioventricular canal (ostium primum atrial septal defect), with or without atrioventricular valve repair	CHD
CPT4	33665	Repair of intermediate or transitional atrioventricular canal, with or without atrioventricular valve repair	CHD
CPT4	33670	Repair of complete atrioventricular canal, with or without prosthetic valve	CHD
CPT4	33675	Closure of multiple ventricular septal defects;	CHD
CPT4	33676	Closure of multiple ventricular septal defects; with pulmonary valvotomy or infundibular resection (acyanotic)	CHD
CPT4	33677	Closure of multiple ventricular septal defects; with removal of pulmonary artery band, with or without gusset	CHD
CPT4	33681	Closure of single ventricular septal defect, with or without patch;	CHD
CPT4	33684	Closure of single ventricular septal defect, with or without patch; with pulmonary valvotomy or infundibular resection (acyanotic)	CHD
CPT4	33688	Closure of single ventricular septal defect, with or without patch; with removal of pulmonary artery band, with or without gusset	CHD
CPT4	33692	Complete repair tetralogy of Fallot without pulmonary atresia;	CHD
CPT4	33694	Complete repair tetralogy of Fallot without pulmonary atresia; with transannular patch	CHD
CPT4	33697	Complete repair tetralogy of Fallot with pulmonary atresia including construction of conduit from right ventricle to pulmonary artery and closure of ventricular septal defect	CHD
CPT4	33702	Repair sinus of Valsalva fistula, with cardiopulmonary bypass;	CHD
CPT4	33710	Repair sinus of Valsalva fistula, with cardiopulmonary bypass; with repair of ventricular septal defect	CHD
CPT4	33720	Repair sinus of Valsalva aneurysm, with cardiopulmonary bypass	CHD
CPT4	33722	Closure of aortico-left ventricular tunnel	CHD

CPT4	33732	Repair of cor triatriatum or supravalvular mitral ring by resection of left atrial membrane	CHD
CPT4	33735	Atrial septectomy or septostomy; closed heart (Blalock-Hanlon type operation)	CHD
CPT4	33736	Atrial septectomy or septostomy; open heart with cardiopulmonary bypass	CHD
CPT4	33737	Atrial septectomy or septostomy; open heart, with inflow occlusion	CHD
CPT4	33770	Repair of transposition of the great arteries with ventricular septal defect and subpulmonary stenosis; without surgical enlargement of ventricular septal defect	CHD
CPT4	33774	Repair of transposition of the great arteries, atrial baffle procedure (eg, Mustard or Senning type) with cardiopulmonary bypass;	CHD
CPT4	33776	Repair of transposition of the great arteries, atrial baffle procedure (eg, Mustard or Senning type) with cardiopulmonary bypass; with closure of ventricular septal defect	CHD
CPT4	33780	Repair of transposition of the great arteries, aortic pulmonary artery reconstruction (eg, Jatene type); with closure of ventricular septal defect	CHD
CPT4	33782	Aortic root translocation with ventricular septal defect and pulmonary stenosis repair (ie, Nikaidoh procedure); without coronary ostium reimplantation	CHD
CPT4	33783	Aortic root translocation with ventricular septal defect and pulmonary stenosis repair (ie, Nikaidoh procedure); with reimplantation of 1 or both coronary ostia	CHD
CPT4	33786	Total repair, truncus arteriosus (Rastelli type operation)	CHD
CPT4	33813	Obliteration of aortopulmonary septal defect; without cardiopulmonary bypass	CHD
CPT4	33814	Obliteration of aortopulmonary septal defect; with cardiopulmonary bypass	CHD
CPT4	33920	Repair of pulmonary atresia with ventricular septal defect, by construction or replacement of conduit from right or left ventricle to pulmonary artery	CHD
CPT4	33361	Transcatheter aortic valve replacement (TAVR/TAVI) with prosthetic valve; percutaneous femoral artery approach	VHD
CPT4	33362	Transcatheter aortic valve replacement (TAVR/TAVI) with prosthetic valve; open femoral artery approach	VHD
CPT4	33363	Transcatheter aortic valve replacement (TAVR/TAVI) with prosthetic valve; open axillary artery approach	VHD
CPT4	33364	Transcatheter aortic valve replacement (TAVR/TAVI) with prosthetic valve; open iliac artery approach	VHD
CPT4	33365	Transcatheter aortic valve replacement (TAVR/TAVI) with prosthetic valve; transaortic approach (e.g., median sternotomy, mediastinotomy)	VHD
CPT4	33366	Transcatheter aortic valve replacement (TAVR/TAVI) with prosthetic valve; transapical exposure<br>(e.g., left thoracotomy)	VHD
CPT4	33367	Transcatheter aortic valve replacement (TAVR/TAVI) with prosthetic valve; cardiopulmonary bypass support with percutaneous peripheral arterial and venous cannulation (e.g., femoral vessels) (List separately in addition to code for primary procedure)	VHD
CPT4	33368	Transcatheter aortic valve replacement (TAVR/TAVI) with prosthetic valve; cardiopulmonary bypass support with open peripheral arterial and venous cannulation (e.g., femoral, iliac, axillary vessels) (List separately in addition to code for primary procedure)	VHD
CPT4	33369	Transcatheter aortic valve replacement (TAVR/TAVI) with prosthetic valve; cardiopulmonary bypass support with central arterial and venous cannulation (e.g., aorta, right atrium, pulmonary artery) (List separately in addition to code for primary procedure)	VHD
CPT4	33390	Valvuloplasty, aortic valve, open, with cardiopulmonary bypass; simple (ie, valvotomy, debridement, debulking and/or simple commissural resuspension)	VHD
CPT4	33391	Valvuloplasty, aortic valve, open, with cardiopulmonary bypass; complex (eg, leaflet extension, leaflet resection, leaflet reconstruction or annuloplasty)	VHD
CPT4	33400	Aortic valvuloplasty	VHD
CPT4	33401	Open valvuloplasty of aortic valve with inflow occlusion	VHD

CPT4	33403	Valvuloplasty, aortic valve; using transventricular dilation, with cardiopulmonary bypass	VHD
CPT4	33404	LV—aorta conduit	VHD
CPT4	33405	Replacement, aortic valve, with cardiopulmonary bypass; with prosthetic valve other than homograft or stentless valve	VHD
CPT4	33406	Replacement, aortic valve, with cardiopulmonary bypass; with allograft valve (freehand)	VHD
CPT4	33410	Replacement, aortic valve, with cardiopulmonary bypass; with stentless tissue valve	VHD
CPT4	33411	Replacement, aortic valve; with aortic annulus enlargement, noncoronary sinus	VHD
CPT4	33412	Replacement, aortic valve; with transventricular aortic annulus enlargement (Konno procedure)	VHD
CPT4	33413	Replacement, aortic valve; by translocation of autologous pulmonary valve with allograft replacement of pulmonary valve (Ross procedure)	VHD
CPT4	33415	Resection or incision of subvalvular tissue for discrete subvalvular aortic stenosis	VHD
CPT4	33418	Transcatheter mitral valve repair, percutaneous approach, including transseptal	VHD
CPT4	33419	Transcatheter mitral valve repair, percutaneous approach, including transseptal puncture when performed; additional prosthesis(es) during same session (List separately in addition to code for primary procedure)	VHD
CPT4	33420	Valvotomy, mitral valve; closed heart	VHD
CPT4	33422	Valvotomy, mitral valve; open heart, with cardiopulmonary bypass	VHD
CPT4	33425	Valvuloplasty, mitral valve, with cardiopulmonary bypass	VHD
CPT4	33426	Valvuloplasty, mitral valve, with cardiopulmonary bypass; with prosthetic ring	VHD
CPT4	33427	Valvuloplasty, mitral valve, with cardiopulmonary bypass; radical reconstruction, with or without ring	VHD
CPT4	33430	Replacement, mitral valve, with cardiopulmonary bypass	VHD
CPT4	33440	Replacement of aortic valve by translocation of autologous pulmonary valve and transventricular aortic annulus enlargement of left ventricular outflow tract with valved conduit replacement of pulmonary valve	VHD
CPT4	33460	Valvectomy, tricuspid valve, with cardiopulmonary bypass	VHD
CPT4	33463	Valvuloplasty, tricuspid valve; without ring insertion	VHD
CPT4	33464	Valvuloplasty, tricuspid valve; with ring insertion	VHD
CPT4	33465	Replacement, tricuspid valve, with cardiopulmonary bypass	VHD
CPT4	33468	Tricuspid valve repositioning and plication for Ebstein anomaly	VHD
CPT4	33470	Valvotomy, pulmonary valve, closed heart; transventricular	VHD
CPT4	33471	Valvotomy, pulmonary valve, closed heart; via pulmonary artery	VHD
CPT4	33472	Incision of valve at right lower heart chamber, open procedure	VHD
CPT4	33474	Valvotomy, pulmonary valve, open heart, with cardiopulmonary bypass	VHD
CPT4	33475	Replacement, pulmonary valve	VHD
CPT4	33476	Right ventricular resection for infundibular stenosis, with or without commissurotomy	VHD
CPT4	33477	Transcatheter pulmonary valve implantation, percutaneous approach, including prestenting of the valve delivery site, when performed	VHD
CPT4	33496	Repair of non-structural prosthetic valve dysfunction with cardiopulmonary bypass (separate procedure)	VHD
CPT4	33600	Closure of atrioventricular valve (mitral or tricuspid) by suture or patch	VHD
CPT4	33602	Closure of semilunar valve (aortic or pulmonary) by suture or patch	VHD
CPT4	92986	Percutaneous balloon valvuloplasty; aortic valve	VHD

CPT4	92987	Percutaneous balloon valvuloplasty; mitral valve	VHD
CPT4	92990	Percutaneous balloon valvuloplasty; pulmonary valve	VHD
CPT4	93355	Echocardiography, transesophageal (TEE) for guidance of a transcatheter intracardiac or great vessel(s) structural intervention(s) (eg, TAVR, transcatheter pulmonary valve replacement, mitral valve repair, paravalvular regurgitation repair, left atrial ap	VHD
CPT4	93590	Percutaneous transcatheter closure of paravalvular leak; initial occlusion device, mitral valve	VHD
CPT4	93592	Percutaneous transcatheter closure of paravalvular leak; each additional occlusion device (List separately in addition to code for primary procedure)	VHD
CPT4	0256T	Implantation of catheter-delivered prosthetic aortic heart valve; endovascular approach	VHD
CPT4	0257T	Implantation of catheter-delivered prosthetic aortic heart valve; open thoracic approach (eg, transapical, transventricular)	VHD
CPT4	0262T	Implantation of catheter-delivered prosthetic pulmonary valve, endovascular approach	VHD
CPT4	0318T	Implantation of catheter-delivered prosthetic aortic heart valve, open thoracic approach, (eg, transapical, other than transaortic)	VHD
CPT4	0343T	Transcatheter mitral valve repair percutaneous approach including transseptal puncture when performed; initial prosthesis	VHD
CPT4	0345T	Transcatheter mitral valve repair percutaneous approach via the coronary sinus	VHD
CPT4	0483T	Transcatheter mitral valve implantation/replacement (TMVI) with prosthetic valve; percutaneous approach, including transseptal puncture, when performed	VHD
CPT4	0484T	Transcatheter mitral valve implantation/replacement (TMVI) with prosthetic valve; transthoracic exposure (e.g., thoracotomy, transapical)	VHD

2. Individual study cohort descriptions and ethics approvals

Full details on individual study genotyping is reported in the HERMES 2.0 study.

Bio-SHiFT-TRIUMPH[1,2] and Deotinchem Cohort Study

Bio-SHiFT (Serial Biomarker Measurements and New Echocardiographic Techniques in chronic Heart Failure Patients Result in Tailored Prediction of Prognosis) is a prospective, observational study of stable patients with chronic heart failure (CHF). The study was conducted in Erasmus MC, Rotterdam, the Netherlands, and Noordwest Ziekenhuisgroep, Alkmaar, the Netherlands. Patients were recruited during their regular outpatient visits and were in clinically stable condition. Patients were eligible if aged ≥18 years and CHF was diagnosed ≥3 months ago according to the guidelines of the European Society of Cardiology. Both patients suffering from HF with reduced ejection fraction and HF with preserved ejection fraction were included. This study was approved by the medical ethics committee of the Erasmus MC, Rotterdam, the Netherlands, and was conducted in accordance with the Declaration of Helsinki. Written informed consent was obtained from all patients. The study is registered in ClinicalTrials.gov, number NCT01851538. TRIUMPH (TRanslational Initiative on Unique and novel strategies for Management of Patients with Heart failure, NTR1893), is an observational prospective study of patients admitted with acute HF in 14 hospitals in the Netherlands between September 2009 and December 2013. Patients were eligible if aged ≥18 years and if they were hospitalized with decompensation of known chronic HF or newly diagnosed HF. The three other inclusion criteria were: 1) natriuretic peptide levels ≥3 times higher than the upper limit of normal; 2) evidence of sustained systolic or diastolic left ventricular dysfunction; and 3) treatment with intravenous diuretics. Patients with HF that was precipitated by a noncardiac condition, by severe valvular dysfunction without sustained left ventricular dysfunction, or by an acute ST-segment elevation myocardial infarction were excluded. Furthermore, patients scheduled for a coronary revascularization procedure, on a waiting list for heart transplantation, with severe renal failure for which dialysis was needed, or with a coexisting condition with a life expectancy <1 year could not participate. The study was approved by the medical ethics committees of all participating centers. All study participants provided written informed consent.

The Doetinchem Cohort Study is an ongoing prospective study in which participants are re-examined every 5 years. A general population sample of 7,769 males and females aged 20 to 59 years was examined in 1987-91 (first round) in Doetinchem, a town in the eastern part of the Netherlands and re-examined in a second (1993-1997), third (1998-2002), fourth (2003-2007), fifth (2008-2012), and sixth round (2013-2017). This study was approved according to the guidelines of the Helsinki Declaration by the external Medical Ethics Committee of the Netherlands Organization for Applied Scientific Research (first four rounds) and the Medical Ethics Committee of the University of Utrecht (later rounds). All participants provided written informed consent.

BioVU

The BioVU cohort consisted of patients at Vanderbilt University Medical Center who have consented to be a part of the program. Study cohort was derived from EHR data available via VUMC's Synthetic Derivative using the phenotype classifier described in the HERMES Consortium analysis plan. The Synthetic Derivative is the de-identified version of the VUMC

EHR and similar to BioVU, contains DNA and genotype data from clinical blood samples that would otherwise be discarded. The Synthetic Derivative can be used in conjunction with BioVU to identify record sets for genome-phenome analysis. All patients gave signed informed consent for use of blood specimens. BioVU is overseen and approved by the VUMC Institutional Review Board.

The Copenhagen Hospital Biobank Cardiovascular Study and The Danish Blood Donor Study (CHB)[3]

This study is part of the study: "Genetics of cardiovascular disease" – a genome-wide association study on repository samples from Copenhagen Hospital Biobank - the CHB-CVS study. CHB has previously been described. CHB-CVS is a genetic study on patients from CHB with cardiovascular disease identified through the Danish National Patient Registry (NPR). CHB-CVS is approved by the National Committee on Health Research Ethics (NVC 1708829) and the Danish Data Protection Agency (P-2019-93). DBDS is a prospective cohort and biobank study on general health among blood donors. The DBDS has previously been described in detail. The genetic studies within DBDS were approved by the National Committee on Health Research Ethics (NVC 1700407) and the Data Protection Agency (P-2019-99). Cases and controls from CHB-CVS and DBDS were defined as per HERMES analysis plan using ICD-10 codes extracted from the NPR and analyzed collectively.

DCM-Garnier[4]

The GWAS by Garnier et al. consisted of 2,719 DCM cases and 4,440 controls in the discovery cohort, recruited from France (DCM samples : CARDIGENE [n=408], EUROGENE [n=84], and PHRC [n=204]; controls: PP3S study [n=1,084]), Germany (DCM samples : German Heart Institute Berlin cohort [n=987], and EUROGENE study [n=214]; Controls: KORA F4 study [n=3,264]), and Italy (DCM samples : EUROGENE [n=82]; Controls: EUROGENE study [n=92]). The study protocol was approved by local ethics committees, complied with the Declaration of Helsinki, and all patients signed informed consent. Full details are supplied in ref 4.

deCODE Heart Failure Study

The deCODE Icelandic heart failure (HF) sample set included patients diagnosed with HF at Landspitali – The National University Hospital (LUH) in Reykjavik and the Primary Health Care Clinics of the Reykjavik capital region, following ICD-9 and ICD-10 codes for heart failure, as described in this manuscript. Information on exclusion criteria and comorbities comes from the same sources. The controls included population controls from the Icelandic genealogical database and individuals recruited through different genetic studies at deCODE genetics. Individuals of non-Icelandic origin were excluded from the study. The study was approved by the Data Protection Authority of Iceland and the National Bioethics Committee of Iceland (Approvals No. VSNb2015080003-03.01 and VSNb2015030022-03.01 with amendments). All participants donating samples signed informed consents.

DiscovEHR

DiscovEHR is a collaboration between Geisinger and the Regeneron Genetics Center. The population is derived from patients who have previously consented to participate in the Geisinger MyCode Community Health Initiative. MyCode is an IRB-approved research study, and all participants have provided informed consent for broad use of samples for research. Participants are broadly recruited to MyCode from both primary and specialty care clinics across the Geisinger system. Exome sequencing and genome-wide genotyping of samples are performed by Regeneron, and genomic data are linked with Geisinger's long-standing

electronic health record, comprising both inpatient and outpatient records. At the time of this study, data for 144,204 patients were available for analysis. This work was supported by the Regeneron Genetics Center and Geisinger. The Geisinger-MyCode initiative and the DiscovEHR study were approved by the Geissinger Institutional Review Board (2006-0528).

Estonian Biobank (EstBiobank)[5]

The Estonian Biobank (EstBiobank) is a population-based biobank with over 200,000 participants. All biobank participants have signed a broad informed consent form. All EstBiobank participants have been genotyped at the Core Genotyping Lab of the Institute of Genomics, University of Tartu. Phenotypes for the present analysis were defined according to HERMES 2.0 analysis plan, using electronic health records (EHRs) available to EstBiobank. Individuals were phenotyped according to 109 separate ICD-10 diagnosis codes (EHRs from Estonian Health Insurance Fund, epicrises and local hospitals) and/or drug prescriptions based on two separate ATC codes (data from Estonian Health Insurance Fund and self-reported by biobank participants). Information on ICD codes is obtained via regular linking with the national Health Insurance Fund and other relevant databases. Research was conducted in accordance with good ethical standards and was approved by the Estonian Committee of Bioethics and Human Research (1.1-12/1020).

The 100,000 Genomes Project (GeL)

The 100,000 Genomes Project (GeL) is a national UK programme that recruited probands with rare diseases and cancer from clinical centres, together with family members, and performed germline and somatic (for a subset of participants with cancer) whole genome sequencing[6,7]. DCM cases were identified from HPO terms at time of study recruitment, and ICD codes from preceding and subsequent clinical episodes. The 100,000 Genomes Project was reviewed by the National Research Ethics Service (14/EE/1112 and 13/EE/032).

The Genetics of Diabetes Audit and Research Tayside Scotland (GoDARTS)[8]

GoDARTS is a cohort study of 18,306 participants, 10,149 with type 2 diabetes and 8,157 healthy controls at baseline recruited from 1996 to 2009 in Tayside, Scotland. Genetic data are available for 8,564 T2D cases and 4,586 controls. Overall, 53.33% of the cohort are male. The majority of the cohort are Caucasian (99.70%) and the median age at recruitment was 64 years. Patients consented to electronic health record linkage to allow follow-up on mortality, hospitalisations and investigations including echocardiography. The GoDARTS study was approved by the Tayside Committee for Medical Research Ethics.

Myocardial Applied Genomics Network (MAGNet)

MAGNet is a collaborative group of investigators based at Perelman School of Medicine, University of Pennsylvania who use genomic approaches to understand human myocardial disease (http://www.med.upenn.edu/magnet/, NHLBI grant number 1R01HL105993-01A1). The present analysis includes a case-control GWAS comparing a subset of patients with prevalent heart failure referred for evaluation and treatment at a heart failure specialty centre at a local institution. Prevalent heart failure was identified by a heart failure cardiologist based on clinical evaluation and cardiac imaging.

The Mass General Brigham Biobank (MGB)

The Mass General Brigham (formerly “Partners Healthcare”) Biobank is a large research data and sample repository comprising more than 100,000 participants that is embedded within the framework of Mass General Brigham Personalized Medicine. Participants are prospectively enrolled in the context of outpatient visits, inpatient stays, and emergency department

encounters. The Biobank contains banked samples (plasma, serum, DNA and buffy coats), genomic data, and other health information, including data from the electronic health record (EHR) at hospitals affiliated with the Mass General Brigham Healthcare system – primarily the Massachusetts General Hospital and the Brigham and Women's Hospital in Boston, MA. Array- based genotyping was performed using either the Illumina Multi-Ethnic Genotyping Array, Expanded Multi-Ethnic Genotyping Array, or the Multi-Ethnic Global BeadChip Array (Illumina, Inc., San Diego, CA). The present study included the first 25,784 genotyped participants of European genetic ancestry from the Mass General Brigham Biobank with relevant clinical data available. The study was conducted in accordance with the Declaration of Helsinki, and approved by the Institutional Review Board of Mass General Brigham (protocol code 2009-P-002312).

Prospective Investigation of the Vasculature in Uppsala Seniors (PIVUS)[9]

The PIVUS study, initiated in 2002, is a community-based prospective cohort comprising 1016 randomly selected men and women aged 70 years in Uppsala county. Subjects were re-investigated at age 75 and age 80 years. The PIVUS-study is unique in its detailed characterization of vascular and cardiac function and morphology. The cohort is also well characterized with regards to genomics, epigenomics, proteomics and metabolomics. Ten year follow-up data on cardiovascular events and mortality is available. The study was approved by the Ethics Committee of the University of Uppsala and the participants gave informed consent.

The Royal Brompton and Harefield Hospital, UK (RBH)[10]

The Royal Brompton & Harefield Hospitals, UK are tertiary national cardiomyopathy referral centers. Participants with dilated cardiomyopathy were diagnosed using national and international clinical guidelines, with advanced cardiac imaging in all cases, and were recruited into the Royal Brompton & Harefield Hospitals Cardiovascular Research Biobank[11] (RBH). The Royal Brompton Biobank was reviewed and approved by the South Central – Hampshire B Research Ethics Committee (09/H0504/104+5 and 19/SC/0257).

University College of London, UK (UCL) and COVID-sortium

The UCL Dilated Cardiomyopathy Cohort were recruited as part of the Inherited Cardiac Disease: A clinical and genetic investigation study, a research registry of patients referred to specialist cardiomyopathy clinics at the UCLH Heart Hospital and Saint Bartholomew's Hospital. Patients were included who had a clinical diagnosis of DCM (defined as LVEF < 50% in the absence of abnormal loading conditions or coronary artery disease sufficient to cause the phenotype). Sample dates ranged from October 2010 to September 2020. Control participants were healthcare workers recruited to the COVID-sortium Healthcare Workers Bioresource. COVID-sortium was approved by the ethical committee of UK National Research Ethics Service (20/SC/0149) and conforms to the principles of the Helsinki Declaration, with all subjects providing written consent.

UK Biobank (UKB)[12]

The UK Biobank is a population-based prospective cohort with extensive genetic and phenotypic data collected on approximately 500,000 individuals aged 40–69 years recruited from across the UK between 2006 and 2010. Collected information include socio-demographics, lifestyle, and health-related factors, physical measures, biological samples (blood, urine, and saliva) for genomics and biochemical markers assessments, linked electronic health records, disease registers, and death register, with a planned repeat assessments and multi-modal imaging. The UK Biobank genetic data contains genotypes for

488,377 participants assayed using two very similar genotyping arrays with extensive phasing and genotype imputation. To define phenotypes used in the GWAS analysis, the present study used the diagnosis and procedure codes in linked primary care record (covering 228,948 participants with latest clinical event recorded on 18 August 2019) and hospital admission record (covering 435,632 participants with latest diagnosis or procedure code recorded on 30 June 2020). The UK Biobank study was reviewed by the National Research Ethics Service (11/NW/0382, 21/NW/0157).

Uppsala Longitudinal Study of Adult Men (ULSAM)[13]

The ULSAM study is a longitudinal community based cohort study of 50 year old men that started in 1970 (n=2322). All 50 year old men in Uppsala were invited to participate. Subjects were reinvestigated at the ages of 60, 70, 77, 82 and 88 years. GWAS data are available in approximately half of the study sample. Untargeted metabolomics and proteomics data are available both in serum and urine in subsamples of the cohort. A large number of cardiovascular events and mortality data are available with more than 20 years of follow-up. The ULSAM study is described in detail on http://www.pubcare.uu.se/ULSAM. All subjects gave written informed consent and the study was approved by the Ethics Committee of the University of Uppsala.

3. Study level GWAS methods and quality control

The HERMES Consortium recommended individual studies perform GWAS using the following procedure, but did not mandate it. All individual genotyping, quality control (QC), imputation, and analysis methods are reported in **Supplementary Table 2**. Ancestries were defined using self-reported followed by validation using genetically determined ancestry. Pre-imputation quality control (QC) recommendations included per marker (genotyping rate >98%, clear Hardy Weinberg equilibrium deviations and P-values in controls >1x10^{-6}, duplicate concordance, Mendelian inconsistencies, minor allele frequency [MAF] concordance with reference populations, MAF>0.5% or >1%) and per sample (genotyping rate >95%, heterozygosity within 3 standard deviations, exclusion of genetic ancestry outliers on principal component [PC] plots) checks. Related individuals (pihat >0.1) were excluded unless specialized models that account for relatedness (e.g. mixed linear models) were used. Post-QC genotypes were imputed using the Michigan Imputation Server with HRC r1.1 2016 reference. GWAS was performed per study per phenotype, using logistic regression assuming additive genetic effects, adjusted for age, sex, genetic principal components (PC), and study-specific covariates. Additional study-level QC was performed for GWAS summary statistics: variants with >2 alleles, regression coefficients >10, coefficient standard errors >10, minor allele frequency (MAF) <1%, imputation (INFO) score <0.6, or effective sample size <50 were excluded. Of remaining variants, allele frequency differences >0.2 compared against European ancestry reference panel (whole-genome sequencing data from HRC and 1000G project phase 3 version 5) were further excluded. Genomic inflation adjustment were applied for summary statistics with genomic control coefficient >1.1.

4. Mendelian cardiomyopathy causing genes from ClinGen and GenCC

List of genes used to test for enrichment of Mendelian cardiomyopathy genes within GWAS loci (https://www.clinicalgenome.org and https://thegencc.org accessed 1st February 2023).

Gene	Phenotype	ClinGen	GenCC
ACTC1	dilated cardiomyopathy	Moderate	Moderate
ACTC1	hypertrophic cardiomyopathy	Definitive	Definitive
ACTN2	intrinsic cardiomyopathy	Moderate	Moderate
ALPK3	hypertrophic cardiomyopathy	Definitive	Strong
BAG3	dilated cardiomyopathy	Definitive	Definitive
CSRP3	hypertrophic cardiomyopathy	Moderate	Moderate
DES	dilated cardiomyopathy	Definitive	Definitive
DES	arrhythmogenic right ventricular cardiomyopathy	Moderate	Moderate
DSG2	arrhythmogenic right ventricular cardiomyopathy	Definitive	Definitive
DSP	arrhythmogenic cardiomyopathy with woolly hair and keratoderma	Definitive	Strong
FHOD3	hypertrophic cardiomyopathy		Moderate
FLNC	dilated cardiomyopathy	Definitive	Definitive
JPH2	dilated cardiomyopathy	Moderate	Moderate
JPH2	hypertrophic cardiomyopathy	Moderate	Moderate
LMNA	dilated cardiomyopathy	Definitive	Definitive
MYBPC3	hypertrophic cardiomyopathy	Definitive	Definitive
MYH7	dilated cardiomyopathy	Definitive	Definitive
MYH7	hypertrophic cardiomyopathy	Definitive	Definitive
MYL2	hypertrophic cardiomyopathy	Definitive	Definitive
MYL3	hypertrophic cardiomyopathy	Definitive	Strong
NEXN	dilated cardiomyopathy	Moderate	Moderate
PKP2	arrhythmogenic right ventricular cardiomyopathy	Definitive	Definitive
PLN	intrinsic cardiomyopathy	Definitive	Definitive
PLN	arrhythmogenic right ventricular cardiomyopathy	Moderate	Moderate
PRKAG2	hypertrophic cardiomyopathy	Definitive	Definitive
RBM20	dilated cardiomyopathy	Definitive	Definitive
SCN5A	dilated cardiomyopathy	Definitive	Definitive
TNNC1	dilated cardiomyopathy	Definitive	Definitive
TNNC1	hypertrophic cardiomyopathy	Moderate	Moderate
TNNI3	dilated cardiomyopathy	Moderate	Moderate
TNNI3	hypertrophic cardiomyopathy	Definitive	Definitive
TNNT2	dilated cardiomyopathy	Definitive	Definitive
TNNT2	hypertrophic cardiomyopathy	Definitive	Definitive
TPM1	dilated cardiomyopathy	Moderate	Moderate
TPM1	hypertrophic cardiomyopathy	Definitive	Definitive
TTN	dilated cardiomyopathy	Definitive	Definitive
VCL	dilated cardiomyopathy	Moderate	Moderate

5. Curation of pathogenic and likely pathogenic rare variants in DCM-causing genes in the UK Biobank

Variants 100 bp up- and down-stream the region of 19 DCM-associated genes with definitive, strong, or moderate evidence for disease, were extracted from the whole exome sequencing data of 454,756 UKB participants[17]. MANE, protein-altering variants that had a MAF <0.1% in gnomAD and UKBB were identified. Intron variants that were pathogenic in ClinVar were manually curated for functional evidence of splicing. Splice region variants and other splice variants (excluding essential splice and splice donor 5th base), were included if they were predicted to cause splicing through SpliceAI (threshold >0.8)[18]. Only *TTN* truncating variants in PSI>90% exons or variants with a splice prediction or curation of pathogenic (with assessment) were included.

The variant list was then shortened to only include the 12 definitive or strong evidence DCM genes (*BAG3*, *DES*, *DSP*, *FLNC*, *LMNA*, *MYH7*, *PLN*, *RBM20*, *SCN5A*, *TNNC1*, *TNNT2*, *TTN*). LOFTEE was used to identify low confidence LoF variants and identify PAVs that are mislabelled (e.g. NAGNAG sites)[19]. All predicted LoF variants were annotated for prediction of NMD escaping; using the NMD plugin, a threshold of 55bp into the penultimate exon of the genes, and additional curation of frameshift variants upstream to the 55bp threshold but where the PTC is predicted to be within the threshold using HGVSp. Additionally, "missense_variant, splice_region_variant"s were only flagged as NMD escaping if they also had a prediction of splicing by SpliceAI. The variants were then filtered for disease-causing mechanisms; all protein-altering variants were kept for *BAG3*, *LMNA*, *PLN*, *SCN5A*, *RBM20*, and *DSP*; variants influencing gene product structure (i.e. indels, missense, start and stop lost) or gene product level (i.e. frameshift, splice, stop gained) (but NMD escaping) were kept for *MYH7*, *DES*, *TNNC1*, and *TNNT2*; and variants predicted to influence gene product level were kept for *TTN* and *FLNC*.

The variant list was then shortened to only include variants that met a filtering allele frequency (FAF) (gnomAD population max FAF <0.00004) and as a MAF in UKB. Additionally, compound heterozygous carriers of common TTN truncating variants (same two TTN variants identified in >10 individuals) were removed from the analysis as these were likely rescued. Finally, only variants that would be called pathogenic or likely pathogenic if identified in a patient with DCM were included; using Cardioclassifier and ClinVar, the variants were manually curated if they had any evidence of pathogenicity.

6. Individual study acknowledgements

Bio-SHiFT-TRIUMPH and Doetinchem Cohort Study

The Bio-SHiFT study was supported by the Jaap Schouten Foundation and the Noordwest Academie. TRIUMPH was supported by the framework of the Center for Translational Molecular Medicine, project TRIUMPH (grant 01C-103).

We thank the Dutch Ministry of Health, Welfare and Sport and the National Institute for Public Health and the Environment (RIVM), Bilthoven, the Netherlands, for their contribution and ongoing support to the Doetinchem Cohort Study

BioVU

The dataset(s) used for the analyses described were obtained from Vanderbilt University Medical Center's BioVU which is supported by numerous sources: institutional funding, private agencies, and federal grants. These include the NIH funded Shared Instrumentation Grant S10RR025141; and CTSA grants UL1TR002243, UL1TR000445, and UL1RR024975.

CHB

We would like to thank the patients in CHB-CVDC and the participants in DBDS. We thank deCODE genetics/Amgen and Department of Clinical Immunology, Copenhagen University Hospital for financial support of this study. Furthermore, we thank the staff working in the biobank facilities and in the hospitals whose work make the existence of this cohort sustainable. This work was supported by the Novo Nordisk Foundation (NNF) (grant numbers: NNF17OC0027594, NNF14CC0001 and NNF18SA0034956), Innovation Fund Denmark (grant number: 5153-00002B) and Nordforsk, Precision Medicine Heart (grant number: 90580 PM Heart).

deCODE

We at deCODE thank the women and men of Iceland that have participated in our studies and our colleagues that contributed to data collection and processing.

DiscovEHR

We acknowledge and thank all participants in Geisinger's MyCode Community Health Initiative for their support and permission to use their health and genomic information in the DiscovEHR collaboration. This work was supported by the Regeneron Genetics Center and Geisinger.

EstBiobank

The establishment and maintenance of the Estonian Biobank has been supported by Targeted Financing from the Estonian Ministry of Science and Education [SF0180142s08], the Ministry of Social Affairs and the Ministry of Economic Affairs and Communications; the biobank and the Genome Center have been supported by the Development Fund of the University of Tartu (grant SP1GVARENG); the European Regional Development Fund has supported the Centre of Excellence in Genomics (EXCEGEN; grant 3.2.0304.11-0312) and through FP7 grants [278913, 306031, 313010, 245536, 269213, 212111, 201413].

Garnier-DCM

This work was supported by grants from the GENMED Laboratory of Excellence on Medical Genomics [ANR-10-LABX-0013]; DETECTIN-HF project (ERA-CVD framework); Assistance Publique-Hôpitaux de Paris [PHRC programme hospitalier de recherche Clinique,

AOM04141]; Délégation à la recherche clinique AP-HP [EMUL and PHRCn_AOM95082]; the 'Fondation LEDUCQ' [Eurogene Heart Failure network; 11CVD 01]; the PROMEX charitable foundation and the Société Française de Cardiologie/Fédération Française de Cardiologie. The SFBTR19 registry was supported by the Deutsche Forschungsgemeinschaft (DFG). The KORA study was initiated and financed by the Helmholtz Zentrum München – German Research Center for Environmental Health, funded by the German Federal Ministry of Education and Research (BMBF) and by the State of Bavaria. KORA research was supported at the Munich Center of Health Sciences (MC-Health), Ludwig-Maximilians-Universität, as part of LMUinnovativ.

GeL

This research was made possible through access to the data and findings generated by the 100,000 Genomes Project under Project Number 667. The 100,000 Genomes Project is managed by Genomics England Limited (a wholly owned company of the Department of Health and Social Care), and funded by the National Institute for Health Research and NHS England. The Wellcome Trust, Cancer Research UK and the Medical Research Council have also funded research infrastructure. The 100,000 Genomes Project uses data provided by patients and collected by the National Health Service as part of their care and support. Genotyping was supported by the Institute of Psychiatry Psychology and Neuroscience (IoPPN) Genomics & Biomarker Core Facility within King's College London, who gratefully acknowledge capital equipment funding from the Maudsley Charity [980] and Guy's and St Thomas's Charity [STR130505].

MAGNet

We thank the Broad Institute for generating high-quality sequence data with Patrick Ellinor as the PI. The datasets used in this manuscript were obtained from dbGaP at http://www.ncbi.nlm.nih.gov/gap through dbGaP accession number phs001539.

RBH

This work was supported by funding from the British Heart Foundation [RE/18/4/34215, FS/IPBSRF/22/27059, FS/15/81/31817, FS/ICRF/21/26019, RG/19/6/34387, BC/F/21/220106]; the Medical Research Council [MC_UP_1605/13]; Wellcome Trust [107469/Z/15/Z]; the National Institute for Health Research (NIHR) Imperial College Biomedical Research Centre; NIHR Royal Brompton Cardiovascular Biomedical Research Unit; Sir Jules Thorn Charitable Trust [21JTA]; National Heart Lung Institute Foundation; Royston Centre for Cardiomyopathy Research; Rosetrees Trust. This research has been conducted in part using the UK Biobank Resource under Application Numbers 9922, 15422, 18545, 40616 and 47602.

University College of London, UK (UCL)

The authors are grateful to the patients and their families whose contributions have made this work possible. This study was supported by the National Institute for Health Research University College London Hospitals Biomedical Research Centre.

UK Biobank

UK Biobank was established by the Wellcome Trust medical charity, Medical Research Council, Department of Health, Scottish Government and the Northwest Regional Development Agency. It has also had funding from the Welsh Government, British Heart

Foundation, Cancer Research UK and Diabetes UK. UK Biobank is supported by the National Health Service (NHS). UK Biobank has received additional funding for: genotyping of all 500,000 participants (from the Department of Health, Medical Research Council, British Heart Foundation); for the measurement of biochemistry markers in all 500,000 participants (from the Wellcome Trust, Medical Research Council, British Heart Foundation, Diabetes UK); for imaging 100,000 participants (Wellcome Trust, Medical Research Council, British Heart Foundation) and re-imaging 10,000 participants (Dementia Platform UK), for the whole genome sequencing of the full cohort (Medical Research Council, and for establishing a research analysis platform (Wellcome), and for repeat imaging up to 60,000 participants from the imaging programme (Medical Research Council, CZI and Calico).

ULSAM

This study was supported by grants from Primary Health Care in Uppsala County, Royal Scientific Society Foundation (Kungliga vetenskapssamhällets fond), Swedish Heart Lung Foundation (Hjärt-Lungfonden) and Thuréus Foundation.

7. Consortia Members

All investigators and their affiliations are correct at the time of analysis, listed in alphabetical order.

COVIDsortium Investigators

Hakam Abbass[4], Aderonke Abiodun[4,8], Mashael Alfarih[4], Zoe Alldis[4], Daniel M Altmann[10], Oliver E Amin[7], Mervyn Andiapen[4], Jessica Artico[4], João B Augusto[4], Georgina L Baca[4], Sasha N L. Bailey[1], Anish N Bhuva[4], Alex Boulter[4], Ruth Bowles[4], Rosemary J Boyton[1], Olivia V Bracken[12], Ben O'Brien[4,18], Tim Brooks[3], Natalie Bullock[2], David K Butler[1], Gabriella Captur[5,8], Nicola Champion[4], Carmen Chan[4], Aneesh Chandran[7], David Collier[16], Jorge Couto de Sousa[4], Xose Couto-Parada[4], Teresa Cutino-Moguel[4], Rhodri H Davies[4], Brooke Douglas[5], Cecilia Di Genova[13], Keenan Dieobi-Anene[4], Mariana O Diniz[7], Anaya Ellis[3], Karen Feehan[12], Malcolm Finlay[4], Marianna Fontana[5], Nasim Forooghi[4], Celia Gaier[12], Joseph M Gibbons[2], Derek Gilroy[14], Matt Hamblin[4], Gabrielle Harker[3], Jacqueline Hewson[3], Wendy Heywood[19], Lauren M Hickling[15], Aroon D Hingorani[7], Lee Howes[8], Alun Hughes[8], Gemma Hughes[4], Rebecca Hughes[4,8], Ivie Itua[4], Victor Jardim[4], Wing-Yiu Jason Lee[2], Melaniepetra Jensen[4], Jessica Jones[3], Meleri Jones[2], George Joy[4], Vikas Kapil[4,16], Hibba Kurdi[4,8], Jonathan Lambourne[4], Kai-Min Lin[1], Sarah Louth[5], Mala K Maini[7], Vineela Mandadapu[4], Charlotte Manisty[4,8], Áine McKnight[2], Katia Menacho[4], Celina Mfuko[4], Kevin Mills[19], Oliver Mitchelmore[4], Christopher Moon[3], James C Moon[4,8], Diana Munoz-Sandoval[1], Sam M Murray[1], Mahdad Noursadeghi[7], Ashley Otter[3], Corinna Pade[2], Susana Palma[4], Ruth Parker[17], Kush Patel[4], Babita Pawarova[5], Steffen E Petersen[4], Brian Piniera[4], Franziska P Pieper[1], Daniel Pope[4,16], Mary Prossora[4], Lisa Rannigan[5], Alicja Rapala[8], Catherine J Reynolds[1], Amy Richards[4], Matthew Robathan[16], Joshua Rosenheim[7], Genine Sambile[4], Nathalie M. Schmidt[7], Amanda Semper[3], Andreas Seraphim[4], Mihaela Simion[5], Angelique Smit[5], Michelle Sugimoto[12], Leo Swadling[7], Stephen Taylor[3], Nigel Temperton[13], Stephen Thomas[3], George D Thornton[4,8], Thomas A Treibel[4,8], Art Tucker[4], Jessry Veerapen[4], Mohit Vijayakumar[4], Sophie Welch[4], Theresa Wodehouse[4], Lucinda Wynne[4], Dan Zahedi[17]

1. Department of Infectious Disease, Imperial College London, London, UK.
2. Blizard Institute, Barts and the London School of Medicine and Dentistry, Queen Mary University of London, London, UK.
3. National Infection Service, Public Health England, Porton Down, UK.
4. St Bartholomew's Hospital, Barts Health NHS Trust, London, UK.
5. Royal Free London NHS Foundation Trust, London, UK.
6. James Wigg Practice, Kentish Town, London, UK.
7. Division of Infection and Immunity, University College London, London, UK.
8. Institute of Cardiovascular Science, University College London, UK.
9. Academic Rheumatology, Clinical Sciences, Nottingham City Hospital, Nottingham, UK.
10. Department of Immunology and Inflammation, Imperial College London, London, UK.
11. Lung Division, Royal Brompton and Harefield Hospitals, London, UK.
12. Division of Medicine, University College London, London, UK.
13. Viral Pseudotype Unit, Medway School of Pharmacy, Chatham Maritime, Kent, UK.
14. Centre for Clinical Pharmacology, University College London, London, UK.

15. East London NHS Foundation Trust Unit for Social and Community Psychiatry, Newham Centre for Mental Health, London, UK.
16. William Harvey Research Institute, Queen Mary University of London, London, UK.
17. School of Clinical Medicine, University of Cambridge, Cambridge, UK.
18. German Heart Centre and Charité University, Berlin, Germany.
19. UCL Great Ormond Street Institute of Child Health, London, UK.

DBDS Genomic Consortium Investigators

Karina Banasik[1], Jakob Bay[2], Jens Kjærgaard Boldsen[3], Thorsten Brodersen[2], Søren Brunak[1], Kristoffer Burgdorf[1], Mona Ameri Chalmer[4], Maria Didriksen[5], Khoa Manh Dinh[3], Joseph Dowsett[5], Christian Erikstrup[3,6], Bjarke Feenstra[5,7], Frank Geller[5,7], Daniel Gudbjartsson[8], Thomas Folkmann Hansen[4], Lotte Hindhede[3], Henrik Hjalgrim[9,7], Rikke Louise Jacobsen[5], Gregor Jemec[10], Bitten Aagaard Jensen[11], Katrine Kaspersen[3], Bertram Dalskov Kjerulff[3], Lisette Kogelman[4], Margit Anita Hørup Larsen[5], Ioannis Louloudis[1], Agnete Lundgaard[1], Susan Mikkelsen[3], Christina Mikkelsen[5], Ioanna Nissen[5], Mette Nyegaard[12], Sisse Rye Ostrowski[5,13], Ole Birger Pedersen[2,13], Alexander Pil Henriksen[1], Palle Duun Rohde[12], Klaus Rostgaard[9,7], Michael Schwinn[5], Kari Stefansson[8], Hreinn Stefánsson[8], Erik Sørensen[5], Unnur Þorsteinsdóttir[8], Lise Wegner Thørner[5], Mie Topholm Bruun[14], Henrik Ullum[15], Thomas Werge[16,13], David Westergaard[1]

1. Novo Nordisk Foundation Center for Protein Research, Faculty of Health and Medical Sciences, University of Copenhagen, Copenhagen, Denmark
2. Department of Clinical Immunology, Zealand University Hospital, Køge, Denmark
3. Department of Clinical Immunology, Aarhus University Hospital, Aarhus, Denmark
4. Danish Headache Center, Department of Neurology, Copenhagen University Hospital, Rigshospitalet-Glostrup, Copenhagen, Denmark
5. Department of Clinical Immunology, Copenhagen University Hospital, Rigshospitalet, Copenhagen, Denmark
6. Department of Clinical Medicine, Health, Aarhus University, Aarhus, Denmark
7. Department of Epidemiology Research, Statens Serum Institut, Copenhagen, Denmark
8. deCODE Genetics, Reykjavik, Iceland
9. Danish Cancer Society Research Center, Copenhagen, Denmark
10. Department of Dermatology, Zealand University hospital, Roskilde, Denmark
11. Department of Clinical Immunology, Aalborg University Hospital, Aalborg, Denmark
12. Department of Health Science and Technology, Faculty of Medicine, Aalborg Univeristy, Aalborg, Denmark
13. Department of Clinical Medicine, Faculty of Health and Medical Sciences, University of Copenhagen, Copenhagen, Denmark
14. Department of Clinical Immunology, Odense University Hospital, Odense, Denmark
15. Statens Serum Institut, Copenhagen, Denmark
16. Institute of Biological Psychiatry, Mental Health Centre, Sct. Hans, Copenhagen University Hospital, Roskilde, Denmark

Estonian Biobank Investigators

Karina Banasik[1], Jakob Bay[2], Jens Kjærgaard Boldsen[3], Thorsten Brodersen[2], Søren Brunak[1], Kristoffer Burgdorf[1], Mona Ameri Chalmer[4], Maria Didriksen[5], Khoa Manh Dinh[3], Joseph Dowsett[5], Christian Erikstrup[3,6], Bjarke Feenstra[5,7], Frank Geller[5,7], Daniel Gudbjartsson[8], Thomas Folkmann Hansen[4], Lotte Hindhede[3], Henrik Hjalgrim[9,7], Rikke Louise Jacobsen[5], Gregor Jemec[10], Bitten Aagaard Jensen[11], Katrine Kaspersen[3], Bertram Dalskov Kjerulff[3], Lisette Kogelman[4], Margit Anita Hørup Larsen[5], Ioannis Louloudis[1], Agnete Lundgaard[1], Susan Mikkelsen[3], Christina Mikkelsen[5], Ioanna Nissen[5], Mette Nyegaard[12], Sisse Rye Ostrowski[5,13], Ole Birger Pedersen[2,13], Alexander Pil Henriksen[1], Palle Duun Rohde[12], Klaus Rostgaard[9,7], Michael Schwinn[5], Kari Stefansson[8], Hreinn Stefánsson[8], Erik Sørensen[5], Unnur Þorsteinsdóttir[8], Lise Wegner Thørner[5], Mie Topholm Bruun[14], Henrik Ullum[15], Thomas Werge[16,13], David Westergaard[1]

1. Novo Nordisk Foundation Center for Protein Research, Faculty of Health and Medical Sciences, University of Copenhagen, Copenhagen, Denmark
2. Department of Clinical Immunology, Zealand University Hospital, Køge, Denmark
3. Department of Clinical Immunology, Aarhus University Hospital, Aarhus, Denmark
4. Danish Headache Center, Department of Neurology, Copenhagen University Hospital, Rigshospitalet-Glostrup, Copenhagen, Denmark
5. Department of Clinical Immunology, Copenhagen University Hospital, Rigshospitalet, Copenhagen, Denmark
6. Department of Clinical Medicine, Health, Aarhus University, Aarhus, Denmark
7. Department of Epidemiology Research, Statens Serum Institut, Copenhagen, Denmark
8. deCODE Genetics, Reykjavik, Iceland
9. Danish Cancer Society Research Center, Copenhagen, Denmark
10. Department of Dermatology, Zealand University hospital, Roskilde, Denmark
11. Department of Clinical Immunology, Aalborg University Hospital, Aalborg, Denmark
12. Department of Health Science and Technology, Faculty of Medicine, Aalborg Univeristy, Aalborg, Denmark
13. Department of Clinical Medicine, Faculty of Health and Medical Sciences, University of Copenhagen, Copenhagen, Denmark
14. Department of Clinical Immunology, Odense University Hospital, Odense, Denmark
15. Statens Serum Institut, Copenhagen, Denmark
16. Institute of Biological Psychiatry, Mental Health Centre, Sct. Hans, Copenhagen University Hospital, Roskilde, Denmark

HERMES Consortium Investigators

Bitten Aagaard[1], Erik Abner[2], Lance Adams[3], Peter Almgren[4], Charlotte Andersson[5,6], Krishna G. Aragam[7,8,9], Johan Ärnlöv[10,11], Folkert W. Asselbergs[12,13,14], Geraldine Asselin[15], Anna Axelsson Raja[16], Joshua D. Backman[17], John Baksi[18], Paul J. R. Barton[19,20,18], Traci M. Bartz[21], Kiran J. Biddinger[7,9], Mary L. Biggs[21,22], Heather L. Bloom[23], Eric Boersma[24], Isabelle Bond[25], Jeffrey Brandimarto[26], Michael R. Brown[27], Søren Brunak[28], Hans-Peter Brunner-La Rocca[29], Mie Topholm Bruun[30], Rachel Buchan[19,20,18], Leonard Buckbinder[31], Henning Bundgaard[16], Douglas Cannie[25,32], Thomas P. Cappola[26], David J. Carey[33], Mark D. Chaffin[9], Philippe Charron[34,35], Daniel I. Chasman[36,37], Olympe Chazara[38], Xing Chen[31], Xu Chen[39], Jonathan H. Chung[17], William Chutkow[40], John G.F. Cleland[41,42], James P. Cook[43,44], Stuart A. Cook[19,20], Tomasz Czuba[45], Trégouët David-Alexandre[46,47], Simon de Denus[15,48], Antonio de Marvao[19,20], Abbas Dehghan[49], Graciela E. Delgado[50], Spiros Denaxas[51,12,52,13], Alexander S. Doney[53], Marcus Dörr[54,55], Joseph Dowsett[56], J. Gustav Smith[57,45,58], Marie-Pierre Dubé[15,59], Samuel C. Dudley[60], Michael E. Dunn[61], Patrick T. Ellinor[62,9], Perry Elliott[25], Gunnar Engström[4], Villard Eric[34,35], Christian Erikstrup[63,64], Tõnu Esko[2,9], Eric H. Farber-Eger[65], Ghazaleh Fatemifar[66,12], Stephan B. Felix[54,55], Chris Finan[25], Sarah Finer[67], Ian Ford[68], Francoise Fougerousse[69], René Fouodjio[15], Catherine Francis[19,18], Sophie Garnier[34,35], Mohsen Ghanbari[70], Sahar Ghasemi[71,55,72], Jonas Ghouse[73], Vilmantas Giedraitis[74], Franco Giulianini[36], John S. Gottdiener[75], Stefan Gross[54,55], Daníel F. Guðbjartsson[76,77], Hongsheng Gui[78], Karl Guo[79], Rebecca Gutmann[80], Sara Hägg[81], Christopher M. Haggerty[33], Brian Halliday[19,18], Åsa K. Hedman[82], Anna Helgadottir[76], Harry Hemingway[12,51], Albert Henry[25,12], Hans Hillege[83], Aroon D. Hingorani[84,25], Hilma Holm[76], Michael V. Holmes[85,86,87], Craig L. Hyde[31], Erik Ingelsson[88,89,90,91], Hanane Issa[12], Jaison Jacob[92], Deleuze Jean-François[93,47,94], J. Wouter Jukema[95,96], Frederick Kamanu[97,98], Isabella Kardys[24], Maryam Kavousi[70], Kay-Tee Khaw[99], Jorge R. Kizer[100], Marcus E. Kleber[50], Lars Køber[101], Andrea Koekemoer[102], Bill Kraus[103], Karoline Kuchenbaecker[104,105], Yi-Pin Lai[31], David Lanfear[78,106], Chim C. Lang[53], Claudia Langenberg[107,108,109], Michael Lee[19], Honghuang Lin[6,110], Lars Lind[111], Cecilia M. Lindgren[112,9,113], Barry London[114], Luca A. Lotta[115], Ruth C. Lovering[25], Brandon D. Lowery[65], Jian'an Luan[109], Steven A. Lubitz[62,9], R. Thomas Lumbers[13,51,12], Patrik Magnusson[81], Anubha Mahajan[113], Anders Malarstig[82,31], Charlotte Manisty[25,116], Douglass Mann[117], Kenneth B. Margulies[26], Nicholas A. Marston[118], Hilary Martin[119], Winfried März[120,121,50], Kathryn McGurk[19,20], John J.V. McMurray[122], Olle Melander[123], Giorgio Melloni[97,98], David Miller[124], James C Moon[125,25], Ify R. Mordi[53], Thomas Morgan[40,126], Michael P. Morley[26], Andrew D. Morris[127], Andrew P. Morris[44,113], Alanna C. Morrison[27], Lori Morton[61], Michael W. Nagle[31], Christopher P. Nelson[102], Christopher Newton-Cheh[7,128,9], Alexander Niessner[129], Teemu Niiranen[130,131], Raymond Noordam[132], Michela Noseda[19], Mahdad Noursadeghi[133], Christoph Nowak[10], Michelle L. O'Donoghue[118], Declan P O'Regan[19,20], Sisse Rye Ostrowski[56,134], Anjali T. Owens[26], Colin N. A. Palmer[135], Antonis Pantazis[18], Guillaume Paré[136,137,138], Helen M. Parry[53], Lavinia Paternoster[139,140], Ole Birger Pedersen[141,134], Markus Perola[131], Louis-Philippe Lemieux Perreault[15], Marie Pigeyre[142,138], Eliana Portilla-Fernandez[70,143], Sanjay K. Prasad[19,18], Bruce M. Psaty[144,145], Kenneth M. Rice[21], Paul M. Ridker[36,37], Simon P. R. Romaine[102], Carolina Roselli[83,9], Jerome I. Rotter[146,147,148], Christian T. Ruff[118], Mark S. Sabatine[118], Neneh Sallah[12], Perttu Salo[131], Veikko Salomaa[131], Nilesh J. Samani[102], Naveed Sattar[149], Jessica van Setten[150], Sonia Shah[151,25], Svati Shah[152,153,103], Alaa A. Shalaby[154], Akshay Shekhar[61], Diane T. Smelser[33], Nicholas L. Smith[155,145,156], Erik Sørensen[157], Doug Speed[158], Sundararajan Srinivasan[135], Kari Stefansson[76,159], Steen Stender[160], David J. Stott[161], Nicholas P. Sunderland[162], Garðar Sveinbjörnsson[76], Per Svensson[163,164], Daniel I. Swerdlow[165,25], Petros Syrris[25], Mari-Liis Tammesoo[2], Jean-Claude Tardif[15,59], Upasana Tayal[19,18], Kent D. Taylor[166], Maris Teder-Laving[2], Alexander Teumer[55,167,72], Pantazis I Theotokis[20], Guðmundur Thorgeirsson[76,168,159], Unnur Thorsteinsdottir[76,159], Christian Torp-Pedersen[169], Vinicius Tragante[76], Thomas A Treibel[125,25], Stella Trompet[95,132], Danny Tuckwell[92], Benoit Tyl[170], Andre G. Uitterlinden[70,143], Henrik Ullum[171], Ana M Valdes[172], Pim van der Harst[83,150], David van Heel[173], Ramachandran S. Vasan[6,174], Felix Vaura[175,176], Abirami Veluchamy[53,135], Monique Verschuuren[177,178], Niek

Verweij[83], Peter M. Visscher[179,180], Christoffer Rasmus Vissing[16], Uwe Völker[55,181], Adriaan A. Voors[83], Marion van Vugt[150], Lars Wallentin[182], Xiaosong Wang[40], Yunzhang Wang[81], James S. Ware[19,183,18,184,9], Nicholas J. Wareham[115], Dawn Waterworth[79], Peter E. Weeke[73], Raul Weiss[185], Quinn Wells[186], Harvey White[187], Kerri L. Wiggins[22], Jemma B. Wilk[31], L. Keoki Williams[78], Heming Xing[40], Xiao Xu[19,20], Jian Yang[179,180], Yifan Yang[26], Laura M. Yerges-Armstrong[79], Bing Yu[27], Faiez Zannad[188], Faye Zhao[92], Jing Hua Zhao[115], Chaoqun Zheng[16], Sean L. Zheng[19,20,18]

1. Department of Clinical Immunology, Aalborg Univeristy Hospital, Aalborg
2. Estonian Genome Center, Institute of Genomics, University of Tartu, Tartu, 51010, Estonia
3. Geisinger Health System, Danville, PA, USA
4. Department of Clinical Sciences, Lund University, Malmö, Sweden
5. Department of Cardiology, Herlev Gentofte Hospital, Herlev Ringvej 57, Herlev, 2650, Denmark
6. National Heart, Lung, and Blood Institute’s and Boston University’s Framingham Heart Study, Framingham, Massachusetts, USA
7. Cardiovascular Research Center, Massachusetts General Hospital, Boston, MA, USA
8. Center for Genomic Medicine, Massachusetts General Hospital, Boston, MA, USA
9. Program in Medical and Population Genetics, The Broad Institute of MIT and Harvard, Cambridge, MA, USA
10. Department of Neurobiology, Care Sciences and Society/ Section of Family Medicine and Primary Care, Karolinska Institutet, Stockholm, Sweden
11. School of Health and Social Sciences, Dalarna University, Falun, Sweden
12. Institute of Health Informatics, University College London, London, England, UK
13. The National Institute for Health Research University College London Hospitals Biomedical Research Centre, University College London, London, England, UK
14. Department of Cardiology, Amsterdam Cardiovascular Sciences, Amsterdam University Medical Centers, Amsterdam, The Netherlands
15. Montreal Heart Institute, Montreal, Canada
16. Department of Cardiology, The Heart Centre, Copenhagen University Hospital, Rigshospitalet, Ingen Lehmanns vej 7, Copenhagen, 2100, Denmark
17. Regeneron Genetics Center, 777 Old Saw Mill River Road, Tarrytown, NY 10591, USA
18. Royal Brompton & Harefield Hospitals, Guy’s and St. Thomas’ NHS Foundation Trust, London, England, UK
19. National Heart & Lung Institute, Imperial College London, London, England, UK
20. MRC London Institute of Medical Sciences, London, England, UK
21. Department of Biostatistics, University of Washington, Seattle, WA, USA
22. Department of Medicine, University of Washington, Seattle, WA, USA
23. Department of Medicine, Division of Cardiology, Emory University Medical Center, Atlanta, GA, USA
24. Department of Cardiology, Erasmus University Medical Center, Rotterdam, The Netherlands
25. Institute of Cardiovascular Science, University College London, London, England, UK
26. Penn Cardiovascular Institute, Perelman School of Medicine, University of Pennsylvania, Philadelphia, PA, USA
27. Department of Epidemiology, Human Genetics, and Environmental Sciences, The University of Texas School of Public Health, Houston, TX, 77030, USA

28. Novo Nordisk Foundation Center for Protein Research, Faculty of Health and Medical Sciences, University of Copenhagen, Copenhagen, Denmark
29. Maastricht University Medical Center, the Netherlands
30. Department of Clinical Immunology, Odense University Hospital, Odense, Denmark
31. Pfizer Worldwide Research & Development, 1 Portland St, Cambridge, MA, USA
32. London, England, UK
33. Department of Molecular and Functional Genomics, Geisinger, Danville, PA, USA
34. UMR-S1166, Research Unit on Cardiovascular Disorders, Metabolism and Nutrition, Team Genomics & Pathophysiology of Cardiovascular Diseases, Sorbonne Université, INSERM, UMR-S1166, Paris, 75013, France
35. ICAN Institute for Cardiometabolism and Nutrition, Paris, 75013, France
36. Division of Preventive Medicine, Brigham and Women's Hospital, Boston, MA, USA
37. Harvard Medical School, Boston, MA, USA
38. Centre for Genomics Research, Discovery Sciences, BioPharmaceuticals R&D, AstraZeneca, Cambridge, UK
39. Department of Medical Epidemiology and Biostatistics, Karolinska Institutet, Stockholm, Sweden.
40. Novartis Institutes for Biomedical Research
41. National Heart and Lung Institute, Imperial College, London, England, UK.
42. Robertson Centre for Biostatistics & Glasgow Clinical Trials Unit, Institute of Health and Wellbeing, University of Glasgow, Glasgow Royal Infirmary, Glasgow, UK
43. Department of Health Data Science, University of Liverpool, Liverpool, England, UK
44. Department of Biostatistics, University of Liverpool, Liverpool, England, UK
45. Department of Molecular and Clinical Medicine, Institute of Medicine, Gothenburg University and Sahlgrenska University Hospital, Gothenburg, Sweden
46. U1219, Univ. Bordeaux, INSERM, BPH, Bordeaux, 33000, France
47. Laboratory of Excellence GENMED (Medical Genomics), France
48. Faculty of Pharmacy, Université de Montréal, Montreal, Canada
49. MRC-PHE Centre for Environment and Health, Department of Epidemiology and Biostatistics, Imperial College London, London, England, UK
50. Vth Department of Medicine (Nephrology, Hypertensiology, Endocrinology, Diabetology, Rheumatology), Medical Faculty of Mannheim, University of Heidelberg, Heidelberg, Germany
51. Health Data Research UK, London, England, UK
52. British Heart Foundation Data Science Centre, London, England, UK
53. Division of Molecular & Clinical Medicine, University of Dundee, Ninewells Hospital and Medical School, Dundee, DD1 9SY, Scotland, UK
54. Department of Internal Medicine B, University Medicine Greifswald, Greifswald, Germany
55. DZHK (German Center for Cardiovascular Research), partner site Greifswald, Greifswald, Germany
56. Department of Clinical Immunology, Copenhagen University Hospital, Rigshospitalet, Copenhagen, Denmark
57. Department of Cardiology, Clinical Sciences, Lund University and Skåne University Hospital, Lund, Sweden
58. Wallenberg Center for Molecular Medicine and Lund University Diabetes Center, Lund University, Lund, Sweden
59. Faculty of Medicine, Université de Montréal, Montreal, Canada
60. Department of Medicine, Cardiovascular Division, University of Minnesota, Minneapolis, MN, USA

61. Cardiovascular Research, Regeneron Pharmaceuticals, Tarrytown, NY, USA
62. Cardiac Arrhythmia Service and Cardiovascular Research Center, Massachusetts General Hospital, Boston, MA, USA
63. Department of Clinical Immunology, Aarhus University Hospital, Aarhus, Denmark
64. Deparment of Clinical Medicine, Health, Aarhus University, Aarhus, Denmark
65. Vanderbilt Institute for Clinical and Translational Research, Vanderbilt University Medical Center, Nashville, TN, USA
66. Health Data Research UK London, University College London, England, UK
67. Centre for Primary Care and Public Health, Wolfson Institute of Population Health, Queen Mary University of London, London, E1 4NS, England, UK
68. Robertson Center for Biostatistics, Institute of Health and Wellbeing, University of Glasgow, Glasgow, Scotland, UK
69. Translational and Clinical Research, Servier Cardiovascular Center for Therapeutic Innovation, Suresnes, France
70. Department of Epidemiology, Erasmus University Medical Center, Rotterdam, The Netherlands
71. Institute of Genetic Epidemiology, Faculty of Medicine and Medical Center, University of Freiburg, Freiburg, Germany
72. Department of Psychiatry and Psychotherapy, University Medicine Greifswald, Greifswald, Germany
73. Department of Cardiology, Rigshospitalet, Copenhagen University Hospital, Denmark
74. Department of Public Health and Caring Sciences, Geriatrics, Uppsala, Sweden
75. Department of Medicine, Division of Cardiology, University of Maryland School of Medicine, Baltimore, MD, USA
76. deCODE genetics/Amgen Inc., Sturlugata 8, Reykjavik, 101, Iceland
77. School of Engineering and Natural Sciences, University of Iceland, Sæmundargata 2, Reykjavik, 101, Iceland
78. Center for Individualized and Genomic Medicine Research, Department of Internal Medicine, Henry Ford Hospital, Detroit, MI, USA
79. Human Genetics, GlaxoSmithKline, Collegeville, PA, USA
80. Division of Cardiovascular Medicine, University of Iowa Carver College of Medicine, Iowa City, IA, USA
81. Department of Medical Epidemiology and Biostatistics, Karolinska Institutet, Stockholm, Sweden
82. Cardiovascular Medicine unit, Department of Medicine Solna, Karolinska Institute, Stockholm, Sweden
83. Department of Cardiology, University Medical Center Groningen, University of Groningen, Groningen, The Netherlands
84. BHF Research Accelerator, University College London, England, UK
85. Clinical Trial Service Unit and Epidemiological Studies Unit, Nuffield Department of Population Health, Big Data Institute, University of Oxford, Oxford, UK
86. Medical Research Council Population Health Research Unit at the University of Oxford, Oxford, UK
87. National Institute for Health Research Oxford Biomedical Research Centre, Oxford University Hospital, Oxford, UK
88. Department of Medical Sciences, Molecular Epidemiology and Science for Life Laboratory, Uppsala University, Uppsala, Sweden.
89. Department of Medicine, Division of Cardiovascular Medicine, Stanford University School of Medicine, Stanford, CA 94305
90. Stanford Cardiovascular Institute, Stanford University, Stanford, CA 94305

91. Stanford Diabetes Research Center, Stanford University, Stanford, CA 94305
92. Novartis Institutes for Biomedical Research, Cambridge, MA, USA
93. Centre National de Recherche en Génomique Humaine (CNRGH), Institut de Biologie François Jacob, CEA, Université Paris-Saclay, Evry, 91057, France
94. Centre d'Etude du Polymorphisme Humain, Fondation Jean Dausset, Paris, France
95. Department of Cardiology, Leiden University Medical Center, Leiden, The Netherlands
96. Einthoven Laboratory for Experimental Vascular Medicine, LUMC, Leiden, The Netherlands
97. Cardiovascular Disease Initiative, The Broad Institute of MIT and Harvard, Cambridge, MA, USA
98. TIMI Study Group, Division of Cardiovascular Medicine, Brigham and Women's Hospital, Harvard Medical School, Boston, MA, USA
99. Department of Public Health and Primary Care, University of Cambridge, Cambridge, CB2 0QQ, UK
100. Cardiology Section, San Francisco Veterans Affairs Health System, and Departments of Medicine, Epidemiology and Biostatistics, University of California San Francisco, CA, USA
101. Department of Cardiology, Nordsjaellands Hospital, Hilleroed, Copenhagen, Denmark
102. Department of Cardiovascular Sciences, University of Leicester and NIHR Leicester Biomedical Research Centre, Glenfield Hospital, Leicester, England, UK
103. Duke Molecular Physiology Institute, Durham, NC, USA
104. Division of Psychiatry, University College London, London, W1T 7NF, England, UK
105. UCL Genetics Institute, University College London, London, WC1E 6BT, England, UK
106. Heart and Vascular Institute, Henry Ford Hospital, Detroit, MI, USA
107. Precision Healthcare University Research Institute, Queen Mary University of London, London, England, UK
108. Computational Medicine, Berlin Institute of Health (BIH) at Charité - Universitätsmedizin Berlin, Berlin, Germany
109. MRC Epidemiology Unit, Institute of Metabolic Science, University of Cambridge, Cambridge, CB2 0QQ, England, UK
110. Department of Medicine, University of Massachusetts Chan Medical School, Worcester, Massachusetts, USA
111. Department of Medical Sciences, Uppsala University, Uppsala, Sweden
112. Big Data Institute at the Li Ka Shing Centre for Health Information and Discovery, University of Oxford, Oxford, England, UK
113. Wellcome Trust Centre for Human Genetics, University of Oxford, Oxford, England, UK
114. Division of Cardiovascular Medicine and Abboud Cardiovascular Research Center, University of Iowa, Iowa City, IA, USA
115. MRC Epidemiology Unit, Institute of Metabolic Science, University of Cambridge School of Clinical Medicine, Cambridge, CB2 0QQ, UK
116. St Bartholomew's Hospital, Barts Heart Centre, UK
117. Center for Cardiovascular Research, Division of Cardiology, Department of Medicine, Washington University School of Medicine, St. Louis, MO, USA
118. TIMI Study Group, Division of Cardiovascular Medicine, Brigham and Women's Hospital, Harvard Medical School, Cambridge, MA, USA
119. Wellcome Sanger Institute, Wellcome Genome Campus, Hinxton, CB10 1RQ, England, UK

120. Clinical Institute of Medical and Chemical Laboratory Diagnostics, Medical University of Graz, Graz, Austria
121. Synlab Academy, Synlab Holding Deutschland GmbH, Mannheim, Germany
122. BHF Cardiovascular Research Centre, University of Glasgow, Scotland, United Kingdom
123. Department of Internal Medicine, Clinical Sciences, Lund University and Skåne University Hospital, Malmö, Sweden
124. Division of Biosciences, University College London, London, England, UK
125. St Bartholomew’s Hospital, Barts Heart Centre
126. Vanderbilt University School of Medicine
127. Usher Institute of Population Health Sciences and Informatics, University of Edinburgh, Edinburgh, Scotland, UK
128. Center for Human Genetic Research, Massachusetts General Hospital, Boston, MA, USA
129. Department of Internal Medicine II, Division of Cardiology, Medical University of Vienna, Vienna, Austria
130. Department of Medicine, Turku University Hospital and University of Turku, Turku, Finland
131. National Institute for Health and Welfare, Helsinki, Finland
132. Section of Gerontology and Geriatrics, Department of Internal Medicine, Leiden University Medical Center, Leiden, The Netherlands
133. National Heart and Lung Institute, Imperial College, London, England, UK
134. Department of Clinical Medicine, Faculty of Health and Medical Sciences, University of Copenhagen, Copenhagen, Denmark
135. Division of Population Health and Genomics, University of Dundee, Ninewells Hospital and Medical School, Dundee, DD1 9SY, Scotland, UK
136. Department of Pathology and Molecular Medicine, McMaster University, Hamilton, ON, Canada
137. Thrombosis and Atherosclerosis Research Institute, Hamilton, ON, Canada
138. Population Health Research Institute, Hamilton, ON, Canada
139. Medical Research Council Integrative Epidemiology Unit, Bristol Medical School, University of Bristol, Bristol, England, UK
140. Population Health Sciences, Bristol Medical School, University of Bristol, Bristol, England, UK
141. Department of Clinical Immunology, Zealand University Hospital, Køge, Denmark
142. Department of Medicine, McMaster University, Hamilton, ON, Canada
143. Department of Internal Medicine, Erasmus University Medical Center, Rotterdam, The Netherlands
144. Cardiovascular Health Research Unit, University of Washington, Seattle, WA, USA
145. Kaiser Permanente Washington Health Research Institute, Kaiser Permanente Washington, Seattle, WA, USA
146. The Institute for Translational Genomics and Population Sciences, Harbor-UCLA Medical Center, Torrance, CA, USA
147. Departments of Pediatrics and Medicine, Harbor-UCLA Medical Center, Torrance, CA, USA
148. Los Angeles Biomedical Research Institute, Harbor-UCLA Medical Center, Torrance, CA, USA
149. BHF Cardiovascular Research Centre, University of Glasgow, Glasgow, Scotland, UK

150. Department of Cardiology, Division of Heart and Lungs, University Medical Center Utrecht, Utrecht, 3584 CX, The Netherlands
151. Institute for Molecular Bioscience, The University of Queensland, Brisbane, Australia
152. Division of Cardiology, Department of Medicine, Duke University Medical Center, Durham, NC, USA
153. Duke Clinical Research Institute, Durham, NC, USA
154. Department of Medicine, Division of Cardiology, University of Pittsburgh Medical Center and VA Pittsburgh HCS, Pittsburgh, PA, USA
155. Department of Epidemiology, University of Washington, Seattle, WA, USA
156. Seattle Epidemiologic Research and Information Center, Department of Veterans Affairs Office of Research & Development, Seattle, WA, USA
157. Department of Clinical Immunology, Copenhagen University Hospital, Rigshospitalet, Blegdamsvej 9, Copenhagen, Denmark
158. Quantitative Genetics and Genomics, Aarhus University, Aarhus, Denmark
159. Department of Medicine, University of Iceland, Sæmundargata 2, Reykjavik, 101, Iceland
160. Department of Clinical Biochemistry, Copenhagen University Hospital, Herlev and Gentofte, Denmark
161. Institute of Cardiovascular and Medical Sciences, College of Medical, Veterinary and Life Sciences, University of Glasgow, United Kingdom
162. Bristol Heart Institute / University Hospitals Bristol & Weston, University of Bristol, Bristol, England, UK
163. Department of Cardiology, Söderjukhuset, Stockholm, Sweden
164. Department of Clinical Science and Education-Södersjukhuset, Karolinska Institutet, Stockholm, Sweden
165. BenevolentAI, London, England, UK
166. Institute for Translational Genomics and Population Sciences, LABiomed and Departments of Pediatrics at Harbor-UCLA Medical Center, Torrance, Calif., USA 90502
167. Institute for Community Medicine, University Medicine Greifswald, Greifswald, Germany
168. Department of Internal Medicine, Division of Cardiology, National University Hospital of Iceland, Eiríksgata 5, Reykjavik, 101, Iceland
169. Department of Epidemiology and Biostatistics, Aalborg University Hospital, Aalborg, Denmark
170. Translational and Clinical Research, Servier Cardiovascular Center for Therapeutic Innovation, 50 rue Carnot 92284 Suresnes, France
171. Statens Serum Institut, Artillerivej 5, Copenhagen, 2300, Denmark
172. Injury, Recovery and Inflammation Sciences, School of Medicine, Nottingham, UK
173. Centre for Genomics and Child Health, Blizard Institute, Queen Mary University of London, London, E1 4AT, England, UK
174. Sections of Cardiology, Preventive Medicine and Epidemiology, Department of Medicine, Boston University Schools of Medicine and Public Health, Boston, Massachusetts, USA
175. Department of Clinical Medicine, University of Turku, Turku, Finland
176. Finnish Institute for Health and Welfare, Helsinki, Finland
177. Department Life Course and Health, Centre for Nutrition, Prevention and Health Services, National Institute for Public Health and the Environment, Bilthoven, The Netherlands
178. Julius Center for Health Sciences and Primary Care, University Medical Center Utrecht, Utrecht, The Netherlands

179. Institute for Molecular Bioscience, The University of Queensland, Brisbane, Queensland 4072, Australia
180. Queensland Brain Institute, The University of Queensland, Brisbane, Queensland 4072, Australia
181. Interfaculty Institute for Genetics and Functional Genomics, University Medicine Greifswald, Greifswald, Germany
182. Uppsala Clinical Research Center, Uppsala University, Uppsala, Sweden
183. MRC London Institute of Medical Sciences, Imperial College London, London, England, UK
184. Hammersmith Hospital, Imperial College Hospitals NHS Trust, London, England, UK
185. Department of Internal Medicine, Division of Cardiovascular Medicine, The Ohio State University Medical Center, Columbus, OH, USA
186. Division of Cardiovascular Medicine, Vanderbilt University, Nashville, TN, USA
187. Green Lane Cardiovascular Service, Auckland City Hospital, USA
188. Université de Lorraine, CHU de Nancy, Inserm and INI-CRCT (F-CRIN), Institut Lorrain du Coeur et des Vaisseaux, Vandoeuvre Lès Nancy, 54500, France

Supplementary Information References

1. van Vark, L.C. *et al.* Prognostic Value of Serial ST2 Measurements in Patients With Acute Heart Failure. *J Am Coll Cardiol* **70**, 2378-2388 (2017).
2. Verschuren, W.M., Blokstra, A., Picavet, H.S. & Smit, H.A. Cohort profile: the Doetinchem Cohort Study. *Int J Epidemiol* **37**, 1236-41 (2008).
3. Ina, H.L. *et al.* Cohort profile: Copenhagen Hospital Biobank - Cardiovascular Disease Cohort (CHB-CVDC): Construction of a large-scale genetic cohort to facilitate a better understanding of heart diseases. *BMJ Open* **11**, e049709 (2021).
4. Garnier, S. *et al.* Genome-wide association analysis in dilated cardiomyopathy reveals two new players in systolic heart failure on chromosomes 3p25.1 and 22q11.23. *Eur Heart J* **42**, 2000-2011 (2021).
5. Leitsalu, L. *et al.* Cohort Profile: Estonian Biobank of the Estonian Genome Center, University of Tartu. *International Journal of Epidemiology* **44**, 1137-1147 (2014).
6. Caulfield, M. *et al.* The National Genomics Research and Healthcare Knowledgebase. (figshare, 2019).
7. Smedley, D. *et al.* 100,000 Genomes Pilot on Rare-Disease Diagnosis in Health Care - Preliminary Report. *N Engl J Med* **385**, 1868-1880 (2021).
8. Hébert, H.L. *et al.* Cohort Profile: Genetics of Diabetes Audit and Research in Tayside Scotland (GoDARTS). *International journal of epidemiology* **47**, 380-381j (2018).
9. Lind, L., Fors, N., Hall, J., Marttala, K. & Stenborg, A. A comparison of three different methods to evaluate endothelium-dependent vasodilation in the elderly: the Prospective Investigation of the Vasculature in Uppsala Seniors (PIVUS) study. *Arterioscler Thromb Vasc Biol* **25**, 2368-75 (2005).
10. Tayal, U. *et al.* Precision Phenotyping of Dilated Cardiomyopathy Using Multidimensional Data. *Journal of the American College of Cardiology* **79**, 2219-2232 (2022).
11. Walsh, R. *et al.* Reassessment of Mendelian gene pathogenicity using 7,855 cardiomyopathy cases and 60,706 reference samples. *Genet Med* **19**, 192-203 (2017).
12. Bycroft, C. *et al.* The UK Biobank resource with deep phenotyping and genomic data. *Nature* **562**, 203-209 (2018).
13. Ingelsson, E., Arnlöv, J., Sundström, J. & Lind, L. The validity of a diagnosis of heart failure in a hospital discharge register. *Eur J Heart Fail* **7**, 787-91 (2005).
14. Aung, N. *et al.* Genome-Wide Analysis of Left Ventricular Image-Derived Phenotypes Identifies Fourteen Loci Associated With Cardiac Morphogenesis and Heart Failure Development. *Circulation* **140**, 1318-1330 (2019).
15. Pirruccello, J.P. *et al.* Analysis of cardiac magnetic resonance imaging in 36,000 individuals yields genetic insights into dilated cardiomyopathy. *Nature Communications* **11**, 2254 (2020).
16. Tadros, R. *et al.* Shared genetic pathways contribute to risk of hypertrophic and dilated cardiomyopathies with opposite directions of effect. *Nature Genetics* **53**, 128-134 (2021).
17. Jordan, E. *et al.* Evidence-Based Assessment of Genes in Dilated Cardiomyopathy. *Circulation* **144**, 7-19 (2021).
18. Jaganathan, K. *et al.* Predicting Splicing from Primary Sequence with Deep Learning. *Cell* **176**, 535-548.e24 (2019).
19. Karczewski, K.J. *et al.* The mutational constraint spectrum quantified from variation in 141,456 humans. *Nature* **581**, 434-443 (2020).
