# Supplementary material for: Genome-wide association analysis provides insights into the molecular etiology of dilated cardiomyopathy
Source: Nat Genet. 2024 Nov 21;56(12):2646–58. doi: 10.1038/s41588-024-01952-y (PMC11631752; doi:10.1038/s41588-024-01952-y)
Supplement: Supplementary file 2 — Reporting Summary [file 41588_2024_1952_MOESM2_ESM.pdf]

nature portfolio

Corresponding author(s): R Thomas Lumbers

Last updated by author(s): Apr 28, 2024

Reporting Summary

Nature Portfolio wishes to improve the reproducibility of the work that we publish. This form provides structure for consistency and transparency in reporting. For further information on Nature Portfolio policies, see our Editorial Policies and the Editorial Policy Checklist.

Statistics

For all statistical analyses, confirm that the following items are present in the figure legend, table legend, main text, or Methods section.

n/a	Confirmed	
☐	☒	The exact sample size (*n*) for each experimental group/condition, given as a discrete number and unit of measurement
☐	☒	A statement on whether measurements were taken from distinct samples or whether the same sample was measured repeatedly
☐	☒	The statistical test(s) used AND whether they are one- or two-sided<br>*Only common tests should be described solely by name; describe more complex techniques in the Methods section.*
☐	☒	A description of all covariates tested
☐	☒	A description of any assumptions or corrections, such as tests of normality and adjustment for multiple comparisons
☐	☒	A full description of the statistical parameters including central tendency (e.g. means) or other basic estimates (e.g. regression coefficient) AND variation (e.g. standard deviation) or associated estimates of uncertainty (e.g. confidence intervals)
☐	☒	For null hypothesis testing, the test statistic (e.g. *F*, *t*, *r*) with confidence intervals, effect sizes, degrees of freedom and *P* value noted<br>*Give P values as exact values whenever suitable.*
☒	☐	For Bayesian analysis, information on the choice of priors and Markov chain Monte Carlo settings
☒	☐	For hierarchical and complex designs, identification of the appropriate level for tests and full reporting of outcomes
☐	☒	Estimates of effect sizes (e.g. Cohen's *d*, Pearson's *r*), indicating how they were calculated

Our web collection on statistics for biologists contains articles on many of the points above.

Software and code

Policy information about availability of computer code

Data collection	No software was used for data collection.
Data analysis	GWAS meta-analysis was performed centrally using METAL v2020-05-05. SNP-based heritability was calculated using LDAK SumHer software v5.2. Genetic correlation between traits was performed using LDSC v1.0.1. Multitrait analysis of GWAS was performed using mtag v1.0.8. Conditionally independent variants were identified using GCTA software v1.92.4. Prioritisation of causal variants was performed using fine-mapping with PolyFun v2020-11-14 and SuSiE v0.11.92. Polygenic prediction score (PoPS) v0.1, OpenTargets Variant2Gene v1.1, S-MultiXcan v0.7.3, and coloc R package v5.2.3 were used for effector gene prioritisation. Rare variant burden testing was performed using Regenie v3.2.4. Pathway enrichment of prioritised genes was performed using g:profiler v0.2.3 package in R. Intercellular communication was performed using CellChat v1.0 package in R, and differential gene expression performed using edgeR v3.32.1 package in R. Tissue-based and cell-type heritability estimation was performed using S-LDSC v1.0.1. Polygenic scores were derived using PRS-CS auto v1.0, with individual PGS scores generated using PLINK v1.9. Phenome-wide association study of PGS was performed using phewas v2018-03-12 package in R.

For manuscripts utilizing custom algorithms or software that are central to the research but not yet described in published literature, software must be made available to editors and reviewers. We strongly encourage code deposition in a community repository (e.g. GitHub). See the Nature Portfolio guidelines for submitting code & software for further information.

Data

Policy information about availability of data

All manuscripts must include a data availability statement. This statement should provide the following information, where applicable:

- Accession codes, unique identifiers, or web links for publicly available datasets
- A description of any restrictions on data availability
- For clinical datasets or third party data, please ensure that the statement adheres to our policy

Data from UK Biobank can be requested from the UK Biobank Access Management System. Data from 100,000 Genomes Project can be accessed following application to join the Genomics England Clinical Interpretation Partnership. ClinGen databases are accessible at https://www.clinicalgenome.org and GenCC databases are accessible at https://search.thegencc.org. GWAS summary statistics are available to download from the Cardiovascular Disease Knowledge Portal (https://api.kpndataregistry.org/api/d/Hv5oWo). Regional association plots for all 80 risk loci are available online (https://hermes-dcm-locus.netlify.app). The PGS are available for download on the Polygenic Score Catalog (https://www.pgscatalog.org/) under accession IDs PGS004861 and PGS004862. The raw single nuclei gene expression dataset is available for download from the European Phenome-Genome Archive (Dataset ID EGAD00001009292).

Human research participants

Policy information about studies involving human research participants and Sex and Gender in Research.

Reporting on sex and gender	The article uses the term sex when referring to biological attribute, and was determined using genetic sex where available. Sex was included as a covariate in all multivariate analyses. Findings are relevant to both male and females.
Population characteristics	Population characteristics include age, sex, ancestry (self-reported and genetic) and genetic principal components for all individuals. Blood pressure and body surface area was available for individuals in the cardiac magnetic resonance imaging substudy of the UK Biobank. Baseline characteristics for cohorts are reported in Supplementary Table 1.
Recruitment	Participants were recruited to the UK Biobank from a large number of national sources (e.g. GP, leaflets and advertising, hospitals, and recruitment drives in the community), and targeted individuals from middle age onwards. 100,000 Genomes Project recruited patients with rare disease and cancer along with their relatives, from clinical centres, initially with an emphasis on genetically unexplained disease. Individual study details on participant recruitment is provided in the Supplementary Methods.
Ethics oversight	All patients gave written informed consent, and all studies were approved by the relevant regional research ethics committees, and adhered to the principles set out in the Declaration of Helsinki. The UK Biobank study was reviewed by the National Research Ethics Service (11/NW/0382, 21/NW/0157). The 100,000 Genomes Project was reviewed by the National Research Ethics Service (14/EE/1112 and 13/EE/032).

Note that full information on the approval of the study protocol must also be provided in the manuscript.

Field-specific reporting

Please select the one below that is the best fit for your research. If you are not sure, read the appropriate sections before making your selection.

☒ Life sciences ☐ Behavioural & social sciences ☐ Ecological, evolutionary & environmental sciences

For a reference copy of the document with all sections, see nature.com/documents/nr-reporting-summary-flat.pdf

Life sciences study design

All studies must disclose on these points even when the disclosure is negative.

Sample size	No sample size calculations were made. We used the maximum number of available cases and controls that passed quality control thresholds/metrics.
Data exclusions	No data were excluded.
Replication	No replication of identified genetic risk loci was performed. This was done to maximise the discovery set. The findings are therefore reported as exploratory, albeit in the context of large sample size numbers. Previous studies from this current group of authors that have used MTAG (Tadros et al. Nat Genet 2020) have subsequently shown in larger sample sizes that replication of loci occurs (Tadros, Zheng et al. Nat Genet under review). Furthermore, there are no additional suitable studies that the authors are aware of to conduct replication in.
Randomization	Observational study - not applicable
Blinding	Observational study - not applicable

Reporting for specific materials, systems and methods

We require information from authors about some types of materials, experimental systems and methods used in many studies. Here, indicate whether each material, system or method listed is relevant to your study. If you are not sure if a list item applies to your research, read the appropriate section before selecting a response.

Materials & experimental systems

n/a	Involved in the study
☒	☐ Antibodies
☒	☐ Eukaryotic cell lines
☒	☐ Palaeontology and archaeology
☒	☐ Animals and other organisms
☒	☐ Clinical data
☒	☐ Dual use research of concern

Methods

n/a	Involved in the study
☒	☐ ChIP-seq
☒	☐ Flow cytometry
☒	☐ MRI-based neuroimaging
